# Predictive Accuracy of Chemotherapy Toxicity Tools in Older Adults with Cancer: A Systematic Review and Diagnostic Test Accuracy Meta-Analysis

**DOI:** 10.3390/cancers18152478

**Published:** 2026-08-02

**Authors:** Edwin Aguirre-Milachay, Mario J. Valladares-Garrido, Nallely V. Chapoñan-Agip, Nelson Luis Cahuapaza-Gutierrez, Betzy C. Torres-Zegarra, Milagros Diaz-Torres, Darwin A. León-Figueroa, Fernando M. Runzer-Colmenares

**Affiliations:** 1 Hospital Nacional Almanzor Aguinaga Asenjo, EsSalud, Chiclayo 14001, Peru; edwinh.aguirre@gmail.com; 2 Escuela de Medicina Humana, Universidad Señor de Sipán, Chiclayo 14001, Peru; vgarrido@uss.edu.pe; 3 Oficina de Inteligencia Sanitaria, Red Prestacional EsSalud Lambayeque, Chiclayo 14001, Peru; 4 CHANGE Research Working Group, Carrera de Medicina Humana, Universidad Científica del Sur, Lima 15001, Peru; 100066763@cientifica.edu.pe (N.V.C.-A.); 100065659@cientifica.edu.pe (N.L.C.-G.); 100066861@cientifica.edu.pe (B.C.T.-Z.); 5 Facultad de Medicina Humana, Universidad de San Martín de Porres, Chiclayo 14001, Peru; milagros_diaz4@usmp.pe; 6 Unidad de Posgrado y Especialización, Tecnologías Avanzadas para la Salud con enfoque en Inteligencia Artificial, Bioinformática y TIC, Universidad Peruana Cayetano Heredia, Lima 15102, Peru; darwin_leon@usmp.pe

**Keywords:** cancer, frail elderly, chemotherapy toxicity, Systematic Review (source: MeSH NLM)

## Abstract

Background: Older adults with cancer are at increased risk of severe treatment-related toxicity. CARG, CRASH, and CARG-BC were developed as toxicity-risk prediction models, whereas G8 was developed as a geriatric screening instrument but has also been evaluated as a predictor of treatment-related toxicity. This study aimed to assess, separately for each instrument, the accuracy with which these tools identify older adults who develop severe chemotherapy-related toxicity. Methods: A systematic review and meta-analysis were conducted in accordance with PRISMA 2020 guidelines. Seven databases were searched through May 2026 for observational studies evaluating the predictive accuracy of the CARG, CRASH, CARG-BC and G8 tools in patients aged ≥65 years initiating chemotherapy. Pooled sensitivity and specificity with 95% confidence intervals (CIs) were calculated, and ROC curves were constructed. Risk of bias was assessed using QUADAS-2 and certainty of evidence was evaluated using GRADE. Results: Twenty-one studies were included, with an overall toxicity prevalence of 52.6%. CARG demonstrated a pooled sensitivity of 79.7% and specificity of 38.3% (AUC = 0.632). CRASH showed sensitivity of 86.9% and specificity of 68.2% (AUC = 0.866), but estimates were based on only four studies. CRASH hematological toxicity showed sensitivity of 75.9% and specificity of 53.1% (AUC = 0.694), with substantial heterogeneity. G8 yielded sensitivity of 69.5% and specificity of 41.5% (AUC = 0.666). CARG-BC showed sensitivity of 83.3% and specificity of 54.4% (AUC = 0.763), based on two breast cancer studies. Certainty of evidence ranged from low to very low. Conclusions: The instruments have distinct purposes and were not pooled against one another. CARG and G8 may be useful for initial risk screening, whereas CRASH showed a more balanced profile but remains supported by limited evidence. None should be used as a stand-alone basis to withhold or modify treatment.

## Simple Summary

Chemotherapy toxicity can substantially affect older adults with cancer. We reviewed 21 observational studies evaluating four risk-assessment approaches: CARG, CRASH, CARG-BC, and G8. The tools CARG, CRASH, and CARG-BC were developed specifically to estimate treatment-toxicity risk, whereas G8 is a geriatric screening tool that has also been studied for its association with severe treatment-related toxicity. Overall toxicity occurred in 52.6% of participants. CARG and G8 may help identify patients who need further assessment, but their low specificity limits their use as stand-alone treatment-decision tools. CRASH showed a more balanced profile, although evidence was based on few studies. These tools should complement comprehensive geriatric assessment and individualized clinical judgment.

## 1. Introduction

Population aging has increased over the years, contributing to a significant increase in the number of older adults with cancer [1]. Currently, about 50% of cancer patients are over 70 years of age, reflecting the high prevalence of this disease in the elderly population [1]. The complexity of cancer biology and its systemic impact requires a comprehensive understanding of the disease, a landscape that is continuously evolving with new diagnostic and therapeutic perspectives [2]. It is estimated that by 2030, approximately 70% of all new cancer diagnoses will occur in people over the age of 65 [3]. This epidemiological shift reflects not only cumulative exposure to carcinogenic factors but also biological processes intrinsic to aging.

Cancer development in older adults is closely linked to chronic low-grade inflammation (“inflammaging”) and immunosenescence. Age-related dysregulation of the immune system promotes a pro-tumorigenic environment characterized by increased levels of proinflammatory cytokines such as interleukin-6 (IL-6), tumor necrosis factor-alpha (TNF-α), and interleukin-1 beta (IL-1β), which contribute to tumor initiation, progression, angiogenesis, and resistance to apoptosis [4,5]. In particular, IL-6 has been consistently associated with worse oncologic outcomes, while TNF-α-mediated activation of NF-κB signaling pathways plays a central role in tumor proliferation and survival [4,5]. Additionally, immunosenescence leads to impaired T-cell and natural killer cell function, reducing immune surveillance and facilitating malignant cell expansion [6]. These biological alterations provide a framework for understanding the increased incidence and complexity of cancer in the elderly population.

Despite these numbers, cancer treatment in older adults remains a significant clinical challenge [1]. Various studies show that these patients can obtain benefits comparable to those of the general population with chemotherapy; however, its use is still lower, both in the adjuvant and metastatic contexts, due to the scant specific evidence and the fear of greater toxicity derived from the progressive decrease in organic functional reserve [6]. Between 30% and 50% of older adults with solid tumors develop severe toxicities (grade 3–5) during treatment [1]. This vulnerability is driven by age-related physiological changes, including reduced organ functional reserve, multimorbidity, frailty, and polypharmacy, all of which significantly influence treatment tolerance and clinical outcomes [6]. In addition, aging modifies pharmacokinetics and pharmacodynamics so that by the age of 80 liver function can be around two-thirds of that of a young adult, increasing the risk of pharmacological toxicity [6].

Given this complexity, chronological age alone is insufficient to guide oncologic decision-making. Therefore, there is a critical need for tools that can accurately predict treatment-related toxicity and support individualized therapeutic strategies. To avoid both undertreatment and overdosing, validated predictive tools based on geriatric assessment have been developed, such as the tool from the Cancer and Ageing Research Group (CARG), the Chemotherapy Risk Assessment Scale for High-Age Patients (CRASH). Additionally CARG-BC was subsequently developed for older adults with early-stage breast cancer. By contrast, G8 was developed to identify patients who may benefit from comprehensive geriatric assessment; nevertheless, several cohorts have evaluated whether an abnormal G8 result is associated with severe treatment-related toxicity [7], and it has been evaluated as a strategy to detect treatment-related complications [8].

Several studies have validated these tools to predict chemotherapy toxicity in older adults with cancer, supporting their usefulness in clinical decision-making [9]. For instance, retrospective analyses have demonstrated significant associations between CARG scores and both hematologic and non-hematologic toxicities [10]. In addition, another Italian study with advanced breast cancer confirmed the usefulness of the CARG and CRASH scales [11]. Although CARG has shown validity in some geographic regions, some studies, such as one in patients with non-small cell lung cancer treated with chemoradiation therapy, found no correlation with grade 3–4 toxicity [12]. In a comparison between the CARG and G8 scales, both were useful in predicting serious toxicity, although CARG demonstrated greater applicability in daily clinical practice [13]. Additionally, these scores and other general and specific ones such as CARG-BC for early breast cancer, MOST score for colon and gastrointestinal cancer, Kanazu for non-small cell lung cancer among others have been evaluated, finding issues with internal and especially external validation in the vast majority of scores, inclusion of treatment-dependent variables but with moderate performance in predicting toxicity [14].

Thanks to medical technological advances, newer chemotherapies, targeted agents, and immunotherapies have been developed, which have become the standard treatment for various types of cancer [9]. However, most of these scales have been validated in specific populations, with low geographical representation and heterogeneity in the types of cancer and treatments used [15]. Moreover, it is unknown to what extent these tools maintain their predictive capacity in patients with comorbidities, geriatric syndromes, or in therapeutic scenarios different from conventional chemotherapy, such as immunotherapy or targeted therapies [15,16], Although new scales like ACTTOP have been developed, which was created for severe toxicity in systemic therapy including chemotherapy targeted therapy, and immunotherapy, there are still independent populations that allow for their analysis [17]. Overall, these limitations highlight an important gap in the literature: the absence of a comprehensive synthesis of the diagnostic performance and comparative utility of chemotherapy toxicity prediction tools in older adults. Therefore, this study aimed to systematically evaluate and synthesize the diagnostic accuracy of severe chemotherapy-related toxicity prediction tools in older adults with cancer, including CARG, CRASH, CRASH hematological toxicity, CARG-BC and G8. Given the heterogeneity in populations, cancer types, treatment regimens, toxicity definitions, and positivity thresholds, pooled estimates were interpreted as summary measures of average performance rather than definitive head-to-head comparisons between tools.

## 2. Materials and Methods

The protocol of this systematic review was registered in PROSPERO [18] (ID: CRD42024588094) and was carried out following the PRISMA 2020 guidelines [19] (Table S1).

### 2.1. Elegibility Criteria

We included observational studies of adults aged ≥65 years with solid or hematological malignancies who were starting or receiving chemotherapy and that evaluated an instrument against severe treatment-related toxicity, usually CTCAE grade 3–5. We distinguished (i) toxicity-specific prediction models (CARG, CRASH, CRASH hematological toxicity, and CARG-BC) from (ii) geriatric screening instruments evaluated for the same target outcome (G8). Quantitative synthesis required at least two independent studies using a sufficiently comparable threshold and providing sensitivity and specificity or data permitting reconstruction of a 2 × 2 table. Instruments supported by a single eligible dataset, instruments developed primarily for broader systemic anticancer therapy without comparable chemotherapy-specific data, and studies lacking reconstructable 2 × 2 data were retained for contextual discussion when relevant but were not pooled. ACTTOP was therefore discussed but not meta-analysed because only one development/validation publication was available and its treatment scope included chemotherapy, targeted therapy, and immunotherapy.

We excluded systematic or narrative reviews, randomised trials, conference proceedings, case reports, editorials, letters, and studies without full-text access. No language restrictions were applied.

### 2.2. Sources of Information and Search Strategy

A comprehensive search was conducted in PubMed, Scopus, Embase, Web of Science, ScienceDirect, the Virtual Health Library, and Google Scholar. The initial search was conducted in August 2024 through December 2024, with monthly updates until May 2026. Search terms included “Drug-Related Side Effects and Adverse Reactions,” “chemotherapy toxicity,” and “frail elderly.” The search was expanded with a second strategy using the keywords: “Antineoplastic Agents”, “Drug-Related Side Effects and Adverse Reactions”, “CARG”, “CRASH”, “CARG-BC”, “G8”, “ACTTOP”, “Anti-Cancer Treatment Toxicity in Older Patients”, “MOST”, “Kanazu”, “CARG-8” until July 2026 without obtaining additional studies beyond those analysed and included. The Rayyan program was used for the evaluation of the articles and the elimination of duplicates before screening [20] (Table S2).

### 2.3. Study Selection

Two independent review authors screened titles and abstracts. We performed a full-text assessment of potentially eligible studies. Disagreements were resolved by discussion and with a third reviewer.

### 2.4. Data Extraction

Two review authors independently extracted data including author, year, country, sample size, age, sex, type and stage of cancer, tool used, toxicity results, and test performance (true positives, false positives, false negatives, true negatives). Discrepancies were resolved by consensus.

### 2.5. Assessing Risk of Bias

The methodological quality of the studies included was assessed using the QUADAS-2 tool, evaluating four domains: patient selection, index test, reference standard, and flow and time. Applicability issues were also assessed [21]. Study-level judgments are reported in Table S5 We also examined whether studies judged at high risk in one or more domains could plausibly influence pooled estimates.

### 2.6. Data Synthesis

For each instrument, risk categories were dichotomized using prespecified clinically used thresholds: CARG 0–5 versus ≥ 6 points; global CRASH 0–6 versus ≥ 7 points; CRASH hematological toxicity 0–3 versus ≥ 4 points; CARG-BC 0–5 versus ≥ 6 points; and G8 > 14 versus ≤ 14 points. These transformations were required to reconstruct 2 × 2 tables but necessarily reduced the granularity of the original multilevel risk scores. Each instrument was meta-analysed separately; estimates from G8 were never statistically pooled with CARG, CRASH, or CARG-BC. Tool-specific sensitivity and specificity with 95% CIs were estimated using bivariate random-effects models. SROC curves were used as descriptive summaries, not as evidence of comparative superiority across tools. Clinical and methodological heterogeneity was assessed from cancer type, study design, geographic region, design study and QUADAS-2 domains. Because several analyses contained fewer than five studies, heterogeneity estimates and non-significant.

For each study, sensitivity and specificity with corresponding 95% confidence intervals (CIs) were calculated. Pooled estimates of sensitivity and specificity were obtained using a random-effects bivariate meta-analysis model, accounting for both within-study variability and between-study heterogeneity. Summary receiver operating characteristic (SROC) curves were generated to evaluate the overall diagnostic performance of the CARG, CRASH, CRASH hem, CARG-BC and G8 scales.

Heterogeneity was assessed using the I^2^ statistic and further explored through meta-regression analyses considering study-level covariates such as age, cancer stage, and geographic region. In addition, the use of a bivariate random-effects model accounts for the correlation between sensitivity and specificity.

In addition, publication bias analysis was performed, obtaining funnel plots and evaluating the *p*-value of the slope coefficient.

Because several tools were evaluated in a small number of studies, meta-regression and heterogeneity estimates were considered exploratory. For analyses with fewer than 10 studies, publication bias assessment was not performed or was interpreted cautiously. Pooled AUC values were not considered universal measures of clinical accuracy, but summary descriptors derived from heterogeneous populations and thresholds.

Finally, we assessed the certainty of the evidence for each outcome using the GRADE (Grading of Recommendations Assessment, Development and Evaluation) methodology. Two review authors independently assessed the certainty of the evidence for each critical outcome as ‘high’, ‘moderate’, ‘low’ or ‘very low’, according to the GRADE approach. Disagreements were resolved by consensus and with the participation of a third reviewer. The Summary of Findings (SoF) table was generated using the GRADEpro GDT (Clinical Practice Guidelines Development Tool) software, and transparent justification for all the judgments made during the evaluation process was described.

## 3. Results

### 3.1. Study Selection

**Figure 1:**
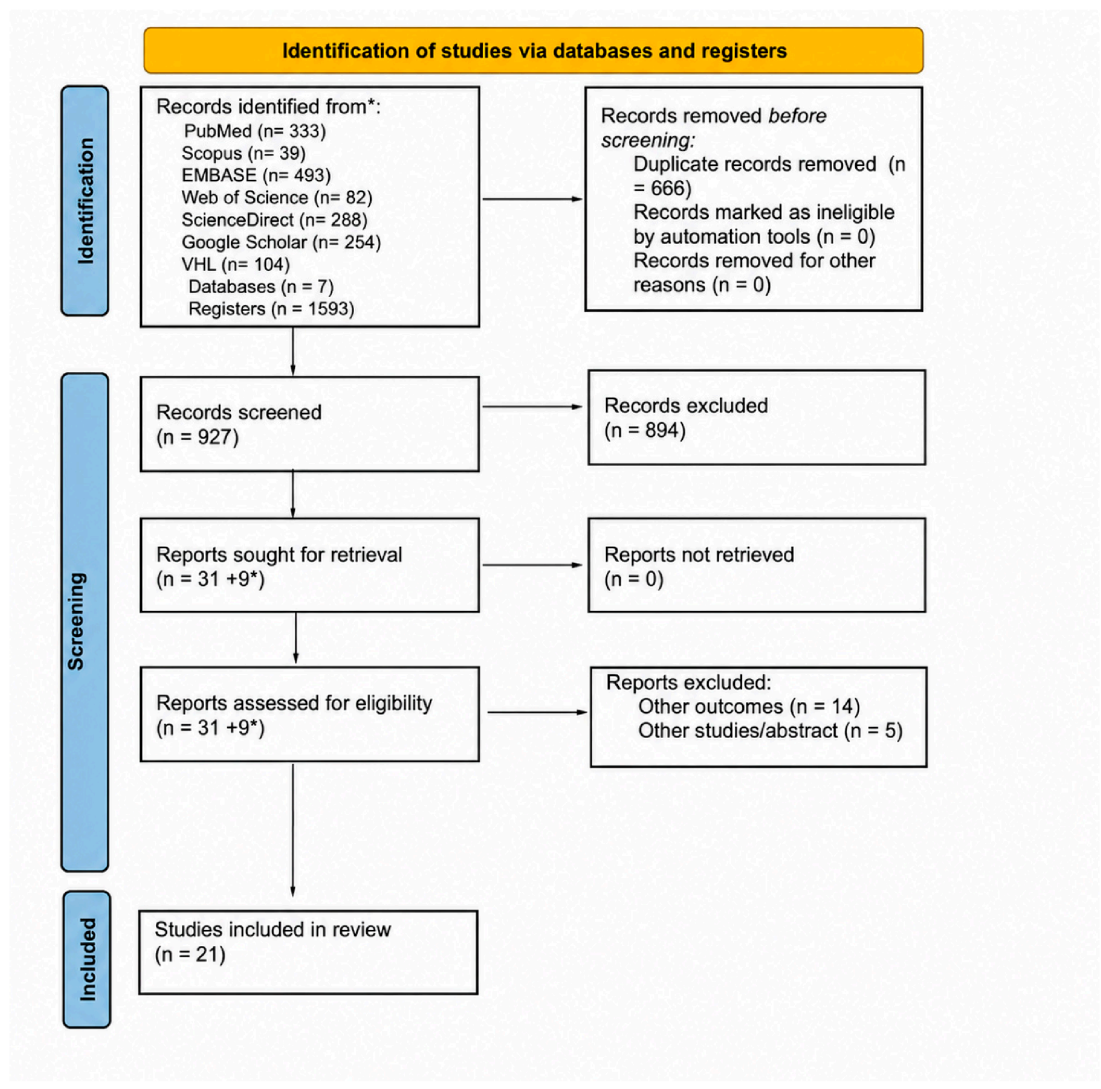
Study selection process. Flow diagram “Preferred Reporting Items for Systematic Reviews and Meta-Analyses”. * The search has been updated until May 2026.

The search strategy yielded 1593 results. After eliminating duplicates, 927 articles were analyzed, contrasting the selection criteria with the titles/abstracts. Then, 40 full-text articles were evaluated and finally 21 studies were included in the systematic review after the updated search through May 2026. The selection process is summarized in Figure 1 using a PRISMA flowchart. In addition, the reasons for exclusion from each study evaluated in full text are detailed in Table S3.

### 3.2. Study Characteristics

We included 21 observational studies published between 2019 and 2024, that were carried out in Canada [22,23,24,25], Japan [26,27,28,29], China [30,31], Iran [32], India [33,34], USA [35,36,37,38,39], Turkey [13], Portugal [40] and Taiwan [41]. In total, 4270 adult patients over 65 years of age with neoplasms were evaluated. Among the 21 studies, 18 included solid neoplasms and 4 included hematological neoplasms, with 51.8% (n = 2193) of the study population being female. The most frequent neoplasm was gastrointestinal type in 30.2% (n = 1257) and the most frequent clinical stages were IV in 19.6% (n = 564) and stage IV in 55% (n = 1582) (Table 1). The most frequent scales used to assess toxicity risk were CARG (n = 16), CRASH (n = 4), CRASH hem (n = 3), CARG-BC (n = 2) and G8 (n = 3). We found only one study that assessed geriatric functionality using Activities of Daily Living, Instrumental Activities of Daily Living, and performance with the BioPsychoSocial model, and one study that assessed grip strength without detailing strength and endurance measurements. We found no predictive studies related to nutritional assessment and sarcopenia.

The prevalence of overall toxicity was 52.6% (n = 2574), while the prevalence of hematologic toxicity reported in studies using the hematologic CRASH scale was 33.9% (n = 204) (Table S4).

### 3.3. Risk of Bias

The methodological quality of the included studies was assessed using the QUADAS-2 tool. Overall, most studies showed low risk of bias in patient selection and reference standard domains.

The measurement result was that nineteen studies presented a low risk in the domain of patient selection, while sixteen studies showed a low risk in the domains of index test, and seven studies in flow, and timing. The main source of potential bias was identified in the flow and timing domain, where several studies showed a high risk of bias, mainly related to incomplete verification or unclear intervals between the index test and the reference standard. Additionally, a low risk was identified in the reference domain across all studies, as all employed the CTCAE classification, suggesting appropriate and reliable diagnostic confirmation in all the included evidence.

**Figure 2:**
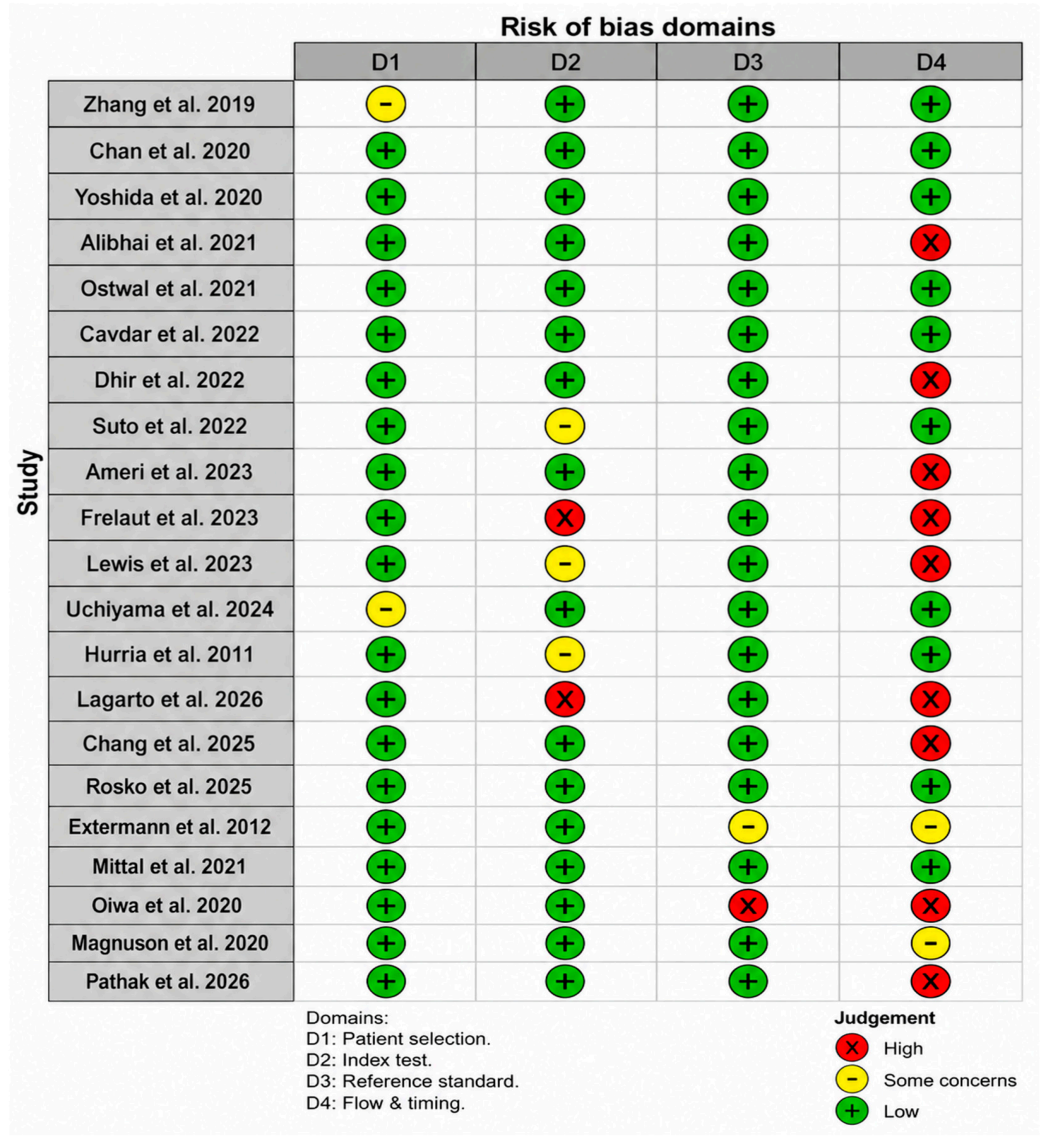
Percentage of studies according to the risk of bias level in each domain of the QUADAS-2 tool [13,22,23,24,25,26,27,28,29,30,31,32,33,34,35,36,37,38,39,40,42].

Regarding concerns about applicability, the majority of the studies were classified as low concern in the domains of patient selection and reference standard, indicating a good generalisation of the findings, with only two studies showing high concern in the index test (Table S5, Figure 2).

### 3.4. Meta-Analysis

#### 3.4.1. CARG Scale

**Figure 3:**
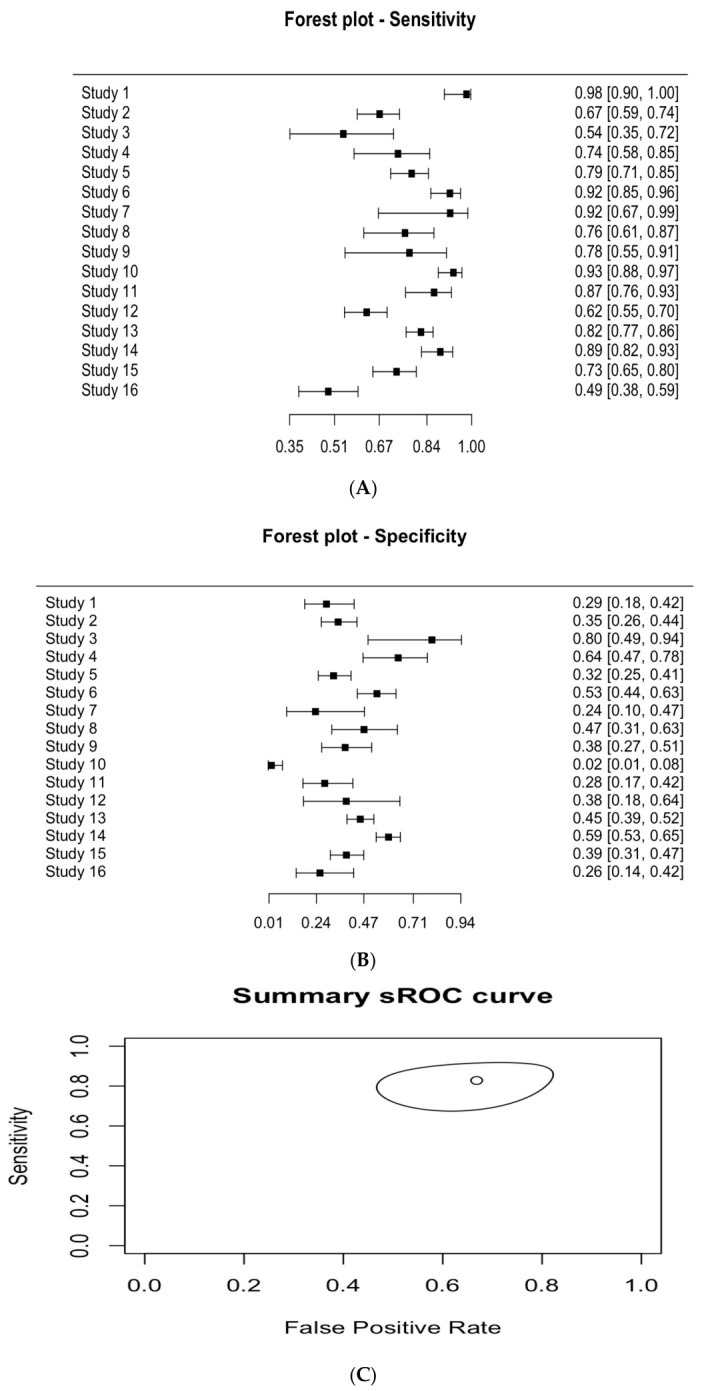
(**A**). Sensitivity meta-analysis of the CARG scale. (**B**). Specificity meta-analysis of the CARG scale. (**C**). ROC curve of the CARG scale.

The results of the 16 studies analyzed by bivariate analysis of random-effects reitsma and REML suggest a mean sensitivity of 79.7% (*p* < 0.001) (Figure 3A)**,** a false positive rate of 61.7% (95% CI: 51.6–70.9%) (*p* < 0.024), and a specificity of 38.3% (Figure 3B) for detecting global grade 3-5 toxicity related to chemotherapy. In addition, the area under the curve (AUC) = 0.632 showed poor diagnostic performance (Figure 3C).

#### 3.4.2. CRASH Scale

**Figure 4:**
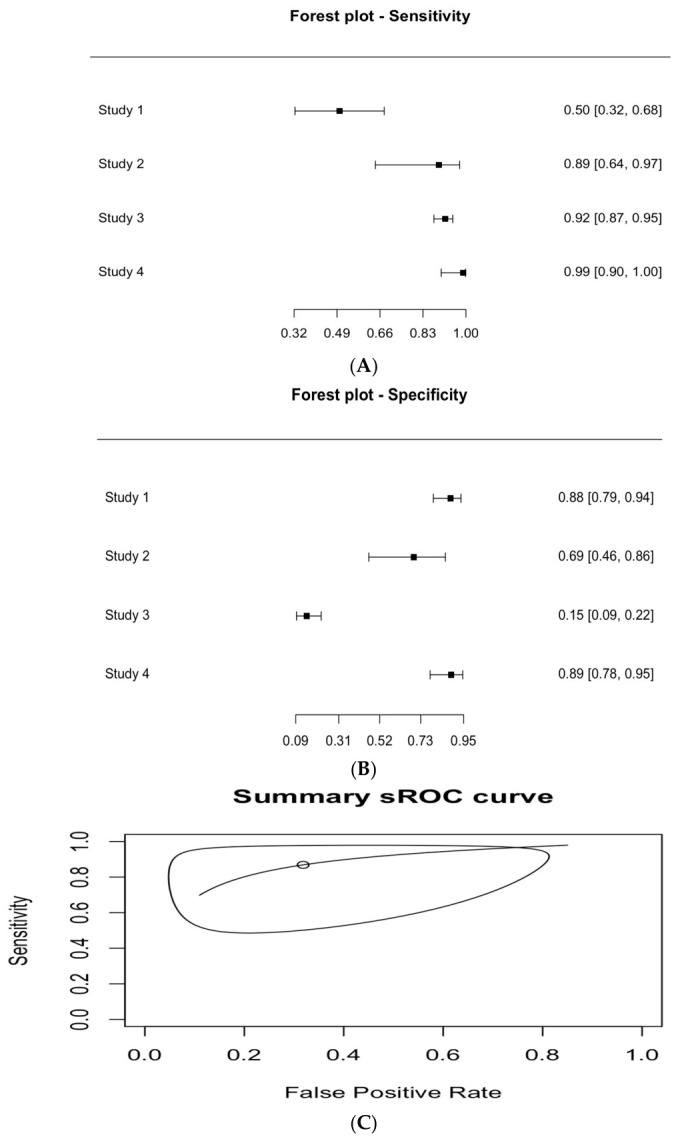
(**A**). Sensitivity meta-analysis of the CRASH scale. (**B**). Specificity meta-analysis of the CRASH scale. (**C**). ROC curve of the CRASH scale.

Meta-analysis of 4 studies found a pooled sensitivity of 86.9% (95% CI: 58.2–96.9%) with statistical significance (*p* = 0.018) (Figure 4A), a false-positive value of 0.318 (95% CI: 0.073–0.736) (*p* = 0.404) and a statistically significant specificity of 68.2%, but with wide confidence intervals (Figure 4B). The AUC of 0.866 indicated a good discriminative ability of the CRASH scale for detecting global grade 3–5 toxicity by chemotherapy (Figure 4C).

#### 3.4.3. CRASH Hem Scale

**Figure 5:**
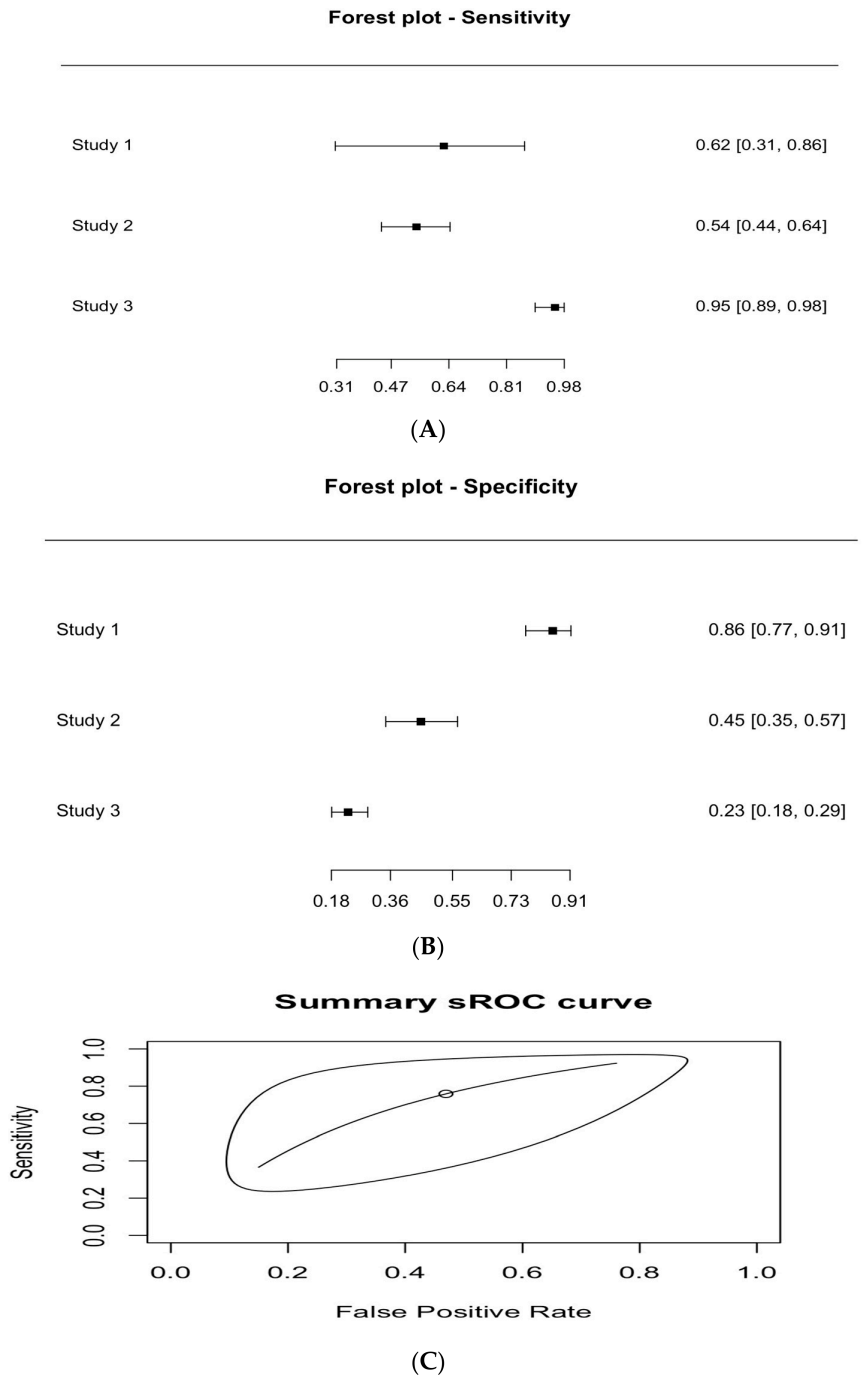
(**A**). Sensitivity meta-analysis of the CRASH hem scale. (**B**). Specificity meta-analysis of the CRASH hem scale. (**C**). ROC curve of the CRASH hem scale.

The meta-analysis of 3 studies showed an overall sensitivity of 75.9% (95% CI: 32.9–95.3%) without statistical significance (*p* = 0.227) (Figure 5A). In addition, the overall specificity was a non-significant 53.1% (*p* = 0.887) (Figure 5B). The AUC (0.694) indicated low performance, estimates were highly heterogeneous (Figure 5C).

#### 3.4.4. G8 Scale

The meta-analysis of 3 studies indicated an overall sensitivity of 69.5% (95% CI: 61.1–76.8%) (*p* < 0.001) (Figure S1). In addition, the true positive rate was 0.585 (95% CI: 0.254–0.854) and the overall specificity was 41.5% without statistical significance (*p* < 0.636) (Figure S2). The AUC (0.666) indicated poor discriminatory capacity (Figure S3).

#### 3.4.5. CARG-BC Scale

The meta-analysis of 2 studies indicated an overall sensitivity of 83.3% (95% CI: 75.2–89.2%) (*p* < 0.001) (Figure S4)**.** In addition, the true positive rate was 0.456 (95% CI: 0.350–0.566) and the overall specificity was 54.4% without statistical significance (*p* < 0.430) (Figure S5). The AUC (0.763) indicated moderate discriminatory capacity (Figure S6).

### 3.5. Heterogeneity and Meta-Regression Analyses

In the analysis of the CARG scale, moderate heterogeneity was observed among the studies, although its magnitude varied substantially depending on the method used to estimate I^2^ (25.2% using the Zhou and Dendukuri approach) (Figure S7). Therefore, heterogeneity should be interpreted as method-dependent and potentially influenced by both clinical variability and statistical instability, rather than being attributed to a single factor such as sample size.

Meta-regression analyses for the CARG scale identified that the study design (diagnostic vs. non-diagnostic) was not significantly associated with sensitivity or the false positive rate. The percentage of patients with stage IV disease was not significantly associated with sensitivity or the false positive rate, although the reduction in I^2^ in this model suggests that differences in disease stage may partially explain the variability between studies (Figures S8 and S9). The geographic region was not significantly associated with diagnostic performance either. Asian studies showed a non-significant trend towards lower sensitivity and a lower false positive rate compared to studies conducted in the Americas, but this finding should be interpreted with caution because the confidence intervals were wide and the analysis was based on study-level data (Figure S10). The high risk of bias study was not significantly associated with sensitivity or false-positive rate. Although high-risk studies showed descriptively higher sensitivity and lower specificity, the estimates were imprecise and residual heterogeneity remained (Figure S11).

Overall, the CARG meta-regression did not identify a robust or statistically consistent source of heterogeneity; rather, the findings suggest that differences in disease stage, region, and study design may contribute to the variability without fully explaining it.

For the CRASH scale, the heterogeneity was substantial, particularly when using the Zhou and Dendukuri approach, which yielded an I^2^ of 71.8%. However, the estimates varied according to the heterogeneity methods, with lower values in the sample size-adjusted approaches. Meta-regression analyses for CRASH should be considered highly exploratory due to the very small number of available studies. Although some models suggested possible associations between study-level variables and diagnostic performance, these findings are unstable and should not be interpreted as definitive evidence of effect modification.

For the CRASH haematological toxicity scale, heterogeneity was high, with an I^2^ of 84.3% using the Zhou and Dendukuri approach and 82.6% to 89.4% using the unadjusted Holling approaches. This indicates a marked inconsistency between the studies and limits the reliability of the pooled estimates. Therefore, the diagnostic performance of CRASH haematological toxicity should be interpreted with caution, especially because the included studies differed in population characteristics, toxicity definitions, and clinical context.

For the G8 scale, the estimates of heterogeneity largely depended on the method used. The Zhou and Dendukuri approach suggested minimal heterogeneity, while the unadjusted Holling approaches indicated very high heterogeneity. Meta-regression suggested that age, sample size, and cancer type may influence sensitivity or false positive rates; however, these analyses were based on a small number of studies and are therefore prone to overfitting and ecological bias. Consequently, these findings should be interpreted as hypothesis-generating and exploratory rather than confirmatory.

For CARG-BC, no heterogeneity was detected in the available studies. However, this finding should not be interpreted as definitive homogeneity because the analysis was based on only two studies. The apparent consistency of CARG-BC is promising, but additional external validation is required before drawing firm conclusions about its generalisability.

Overall, the meta-regression analyses did not provide definitive evidence that age, sample size, disease stage, cancer type, or geographic region consistently explained the heterogeneity between studies. The observed variability is more likely to be multifactorial, reflecting differences in cancer populations, treatment regimens, definitions of toxicity, follow-up duration, study design, and local clinical practice. Therefore, the pooled diagnostic estimates should be interpreted as an average performance in heterogeneous clinical settings rather than universally applicable measures of precision.

### 3.6. Publication Bias

Publication bias assessment was feasible only for the CARG scale, because the remaining tools were evaluated in a limited number of studies. For CARG, no evidence of publication bias or small-study effects was identified. The regression-based assessment showed a non-significant slope coefficient for the association between the log diagnostic odds ratio and the inverse square root of the sample size (*p* = 0.578). Visual inspection of the funnel plot (Figure S12) showed an approximately symmetrical distribution of studies around the regression line, without clear evidence of asymmetry.

Given these findings, no additional correction methods were applied. For the remaining scales, including CRASH, CRASH hematological toxicity, G8, and CARG-BC, publication bias could not be formally assessed because of the small number of available studies. Therefore, the possibility of small-study effects cannot be completely excluded for these tools, and their pooled diagnostic estimates should be interpreted cautiously.

### 3.7. Certainty of the Evidence

The certainty of the evidence was assessed using the GRADE approach for diagnostic test accuracy studies. The Summary of Findings table was updated according to the revised pooled estimates of sensitivity, specificity, and AUC derived from SROC for the CARG, CRASH, CRASH haematological toxicity, G8, and CARG-BC scales. To improve clinical interpretability, the pooled sensitivity and specificity were translated into absolute consequences in a hypothetical population of 1000 older adults receiving chemotherapy.

Overall, the certainty of the evidence ranged from low to very low. For CARG, the certainty was rated as low. Although this scale was supported by the largest number of studies and showed relatively high sensitivity, its specificity was low and there was relevant clinical and methodological heterogeneity among the studies.

For CRASH, the certainty of the evidence was rated as very low. Although the pooled estimates suggested a more balanced diagnostic profile, with higher specificity and good discriminative ability, the evidence was limited by the small number of studies, wide confidence intervals, and substantial heterogeneity. Consequently, CRASH may have potential clinical utility, but the current evidence is insufficient to support definitive conclusions about its comparative superiority.

For the haematological toxicity of CRASH, the certainty of the evidence was also rated as very low. The estimates were unstable, with wide confidence intervals and high heterogeneity, indicating substantial uncertainty regarding their ability to identify older adults at risk of haematological toxicity. Therefore, these results should be interpreted with caution and considered hypothesis-generating.

For G8, the certainty of the evidence was rated as very low. Although G8 showed moderate sensitivity, its specificity was low, resulting in a high number of false positives. This finding is consistent with the original role of G8 as a geriatric screening tool rather than a specific predictive model for toxicity.

For CARG-BC, the certainty of the evidence was rated as low. The scale showed relatively high sensitivity and moderate specificity, with an AUC suggesting moderate discriminative ability. However, only two studies were available, both focused on breast cancer populations, which limits the accuracy and generalisability to other types of tumours. Additional external validation studies are required before firm clinical recommendations can be made (Table 2).

## 4. Discussion

The present meta-analysis synthesized five instrument-specific evidence bases rather than pooling conceptually different tools into a single estimate. CARG, CRASH, CRASH hematological toxicity, and CARG-BC are toxicity-risk prediction models, whereas G8 is a geriatric screening instrument that has been evaluated against severe treatment-related toxicity. The distinction is clinically important: sensitivity and specificity for the same target outcome do not imply that the instruments measure the same construct or should occupy the same position in a clinical pathway.

The CARG scale was the most frequently evaluated tool and therefore provided the largest body of evidence in this review. The original CARG model was developed to predict grade 3–5 chemotherapy-related toxicity in older adults with cancer and was later externally validated in an independent cohort [36,43]. In the present meta-analysis, CARG showed relatively high pooled sensitivity, suggesting that CARG may be useful as an initial screening tool to identify older adults at increased risk of grade 3–5 chemotherapy-related toxicity. However, its specificity was low, and the AUC indicated limited overall discriminative performance. The CARG scale demonstrated the most consistent performance across multiple studies, with a pooled sensitivity that seemed favorable for clinical screening purposes [39]. However, the notably low specificity observed in some studies raises concerns about potential overestimation of toxicity risk, which could lead to unnecessary treatment modifications or patient exclusion from potentially beneficial therapies. Clinically, this means that CARG may help identify patients who require closer monitoring, comprehensive geriatric assessment, optimization of comorbidities, medication review, nutritional support, or chemotherapy dose discussion. Nevertheless, because of its low specificity, CARG should not be used as a stand-alone instrument to deny chemotherapy or exclude patients from potentially beneficial treatment.

These findings align with recent validation studies that have reported conflicting results for the performance of CARG across different populations. Ostwal et al. successfully validated CARG in a cohort of 270 patients with an AUC of 0.63, demonstrating adequate risk discrimination as in the original study (AUC 0.72) [34]. Chan et al. found that CARG presented a weak predictive value in a Chinese population of 259 patients [31]. Similarly, in a German study of 104 patients, Kotzerke et al. reported that CARG demonstrated a strong predictive capacity for severe toxicity (odds ratio [OR] 4.2, 95% CI [1.1, 15.9]) [44], in contrast to the findings of the French ELCAPA cohort in which both CARG and CRASH scores were poor predictors of chemotherapy toxicity [39].

The meta-regression analyses did not identify a statistically robust source of heterogeneity. Study design, percentage of patients with stage IV disease, and geographic region were not significantly associated with diagnostic performance. Although some reductions in heterogeneity were observed in exploratory models, these findings should be interpreted cautiously because they are based on study-level covariates and may be affected by ecological bias. Differences observed between Asian, European, and North American cohorts may reflect not only demographic variability but also differences in healthcare systems, symptom reporting, and treatment practices. These findings underscore the complexity of toxicity prediction in geriatric cancer and highlight the need for scale optimization based on specific patient populations, frailty conditions, and treatment contexts.

In contrast, the CRASH scale showed the most favorable global diagnostic profile among the evaluated tools, with high pooled sensitivity, higher specificity than CARG and G8, and good discriminative ability according to AUC. The original CRASH score was designed to estimate the risk of severe chemotherapy toxicity in older adults and included separate components for hematological and non-hematological toxicity [38]. In the present analysis the global CRASH may offer a more balanced ability to identify older adults who will and will not develop severe chemotherapy toxicity. From a clinical perspective, this balance could make CRASH useful when clinicians need a more specific risk stratification approach, particularly in situations where avoiding unnecessary treatment modification is important. However, these findings must be interpreted with caution because CRASH was evaluated in only a small number of studies, with wide confidence intervals and substantial heterogeneity. Therefore, although CRASH appears promising, the current evidence is insufficient to conclude that it is definitively superior to CARG or other tools [15].

The hematological toxicity component of CRASH showed a different pattern from the global CRASH scale. Its pooled sensitivity and specificity were moderate, and the AUC suggested only limited discrimination. In addition, heterogeneity was high, indicating marked inconsistency between studies. This finding is important because hematological toxicity represents a more specific outcome than global grade 3–5 toxicity and may be influenced by different factors, such as chemotherapy regimen, bone marrow reserve, baseline hemoglobin, renal function, nutritional status, previous treatment exposure, and use of growth-factor support. Therefore, CRASH hematological toxicity should not be interpreted interchangeably with the global CRASH score. Its current role is better understood as a complementary component of toxicity assessment rather than as a reliable stand-alone predictor of hematological complications [38].

The G8 tool showed moderate sensitivity but low specificity and limited discriminative performance. This is consistent with the original design as a geriatric screening tool rather than a specific predictor of toxicity, as noted by Battisti and Arora in their comprehensive review of chemotherapy toxicity prediction tools [9]. The relatively high false-positive rate suggests that G8 may classify many vulnerable patients as being at risk even if they do not develop grade 3–5 toxicity. However, this does not necessarily reduce its clinical usefulness, because the purpose of geriatric screening is to detect vulnerability, frailty, functional decline, malnutrition, or comorbidity burden that may not be captured by oncology performance status alone. Therefore, G8 may be useful as an entry point to geriatric evaluation, but not as a stand-alone tool for chemotherapy dose decisions.

CARG-BC showed high sensitivity, moderate specificity, and moderate SROC AUC. Although this scale was developed for older adults with breast cancer, a population in which treatment decisions are often complex due to competing risks, treatment intent, endocrine options, chemotherapy benefit, and functional reserve [37]. and compared to other tools, a cancer-specific model like the CARG-BC may better capture the variables that are relevant to breast cancer treatment. However, having conducted the analysis with two studies and a non-significant heterogeneity test, the evidence is extremely imprecise; therefore, the absence of detected heterogeneity cannot be interpreted as evidence of consistency. The combined estimate should be considered an exploratory numerical summary accompanied by a narrative interpretation, not a stable basis for clinical recommendations. Therefore, CARG-BC still requires additional external validation before firm clinical recommendations can be made [22].

Other variables, such as cognitive impairment, falls, mobility problems, and comorbidities assessed using various scales, have been associated with an increased risk of severe chemotherapy-related toxicity [45]. Additionally, frailty assessment using indices such as the modified Frailty Index, the Geriatric 8 questionnaire, Adjusted Clinical Groups, the Groningen Frailty Indicator, and comprehensive geriatric assessment are predictors of chemotherapy toxicity in head and neck cancers [46]. Although some functional, cognitive, and nutritional variables are measured by the G8, CARG, CARG-BC and CRASH scales, within the frailty construct they are neither assessed in depth nor holistically; therefore, their predictive value for chemotherapy-related toxicity cannot be determined.

Dichotomising multilevel risk scores enabled reconstruction of common 2 × 2 tables but introduced a loss of prognostic information and potential threshold-related bias. Patients close to a cut-off were treated as categorically different, and variation in the original category definitions could attenuate discrimination or widen uncertainty. The SROC AUCs were derived from heterogeneous studies rather than from a single population evaluated with identical thresholds and outcome definitions. Therefore, AUC should not be interpreted as a universal measure of accuracy or as proof that one scale is superior to another. Recent methodological reviews have emphasized the importance of adequate sample sizes and standardized outcome definitions for the validation of reliable toxicity prediction tools [47,48,49,50]. Methodological guidance for prediction model studies, including TRIPOD and PROBAST, emphasizes the need for transparent reporting, adequate validation, assessment of bias, and evaluation of applicability before clinical implementation [51,52].

On the other hand, although no influence of the study regions on the performance of the CARG test and an explanation for its heterogeneity was found, there is a tendency for the Asian population to exhibit lower sensitivity. The geographic distribution of the studies included reveals important insights into the global applicability of toxicity prediction scales. Studies of Asian populations (China, Japan) demonstrated different performance characteristics compared to North American and European cohorts, indicating potential factors related to ethnicity or health system that influence scale performance. Cross-cultural adaptation studies have shown that geriatric assessment tools require careful validation across different populations, with content validity significantly varying between languages and healthcare systems. Validation of these scales across diverse healthcare settings, from community oncology practices to tertiary academic centers, provides evidence for their broad applicability while highlighting the need for local validation studies. Recent implementation studies have shown that even within the same healthcare system, the performance of a scale can vary significantly between academic centers and community practices, likely reflecting differences in patient populations, resource availability, and clinician experience with geriatric evaluation. Cultural factors, access patterns to healthcare, and treatment protocols may contribute to the variations observed in sensitivity and specificity between different regions. For example, differences in symptom reporting behavior and patient-physician communication styles between cultures may affect the accuracy of patient-reported components within these assessment tools [42,47,53]. Future validation studies should incorporate multicenter designs across diverse healthcare systems, apply standardized definitions of toxicity outcomes, and include formal cross-cultural adaptation processes (including linguistic validation and recalibration of cut-off points). Additionally, external validation in independent cohorts and the evaluation of contextual factors such as healthcare access and treatment patterns are essential to ensure generalizability.

CTCAE remains an essential standardized clinician-reported reference for grading adverse events, but it does not fully represent the frequency, severity, and interference of symptoms experienced by patients. Discordance between clinician- and patient-reported symptomatic toxicity is expected because the two perspectives capture related but distinct information [54,55]. Future research should therefore prespecify the toxicity reference standard and, when feasible, combine CTCAE with PRO-CTCAE to improve ascertainment of symptomatic burden, including lower-grade toxicities that may be clinically important in older adults [56].

The certainty of evidence was low to very low across tools. For CARG, the evidence was rated as low because, despite having the largest number of studies, specificity was limited and heterogeneity remained clinically relevant. For CRASH and CRASH hematological toxicity, the certainty was very low due to the small number of studies, wide confidence intervals, and substantial heterogeneity. For G8, certainty was very low because the tool showed limited specificity and was not originally designed as a toxicity prediction model. For CARG-BC, certainty was low because the estimates were based on only two breast cancer studies, limiting precision and generalizability. These certainty ratings reinforce that the findings should guide risk stratification and future research rather than serve as definitive evidence for treatment decisions.

The present study has limitations. First, the search and quantitative synthesis focused on instruments with at least two comparable chemotherapy-specific datasets and reconstructable 2 × 2 data; newer or treatment-specific models, including ACTTOP, could not be pooled and the review should not be interpreted as an inventory of every existing prediction model. Second, the transformation of multilevel scales into binary outcomes may have introduced threshold-related bias and may have resulted in loss of predictive granularity and attenuation of model performance, potentially contributing to the moderate AUC values and wide confidence intervals observed. Third, the limited number of studies for some tools, particularly CRASH, CARG-BC and G8 reduces the robustness of the pooled estimates. Fourth, the heterogeneity in populations, cancer types, treatment regimens, follow-up periods, and toxicity definitions limits direct clinical generalization. Fifth, several included reports were prognostic validation cohorts rather than classical diagnostic-accuracy studies, which may affect comparability. Sixth, some studies had high risk of bias in flow/timing or index-test domains. Finally, publication bias could only be formally assessed for CARG; therefore, small-study effects cannot be excluded for the remaining tools.

However, the study has strengths. This study provides clinically relevant information by demonstrating that diagnostic performance varies significantly depending on the context, no tool provides consistently high accuracy, differences in sensitivity and specificity of the scales must be considered in clinical use. Therefore, instead of providing a single definitive recommendation, this meta-analysis informs clinicians about the expected variability and limitations of these tools, supporting a more nuanced and individualised decision-making process.

Future research should focus on several key areas to improve the predictive accuracy and clinical applicability of chemotherapy toxicity risk tools in older adults. First, there is a need to standardize toxicity outcome definitions to enhance comparability across studies. Second, future studies should incorporate patient-reported outcomes (PRO-CTCAE) alongside clinician-reported measures to better capture the full spectrum of treatment-related toxicity. Third, the development of hybrid predictive models integrating clinical, functional, and patient-reported variables may improve model performance. Fourth, large-scale multicenter studies across diverse healthcare systems are required to ensure external validity and generalizability. Finally, the increasing use of targeted therapies and immunotherapy introduces new toxicity profiles and biological mechanisms that may not be adequately captured by traditional chemotherapy-based prediction tools. Emerging evidence suggests that predictive biomarkers and tumor-specific characteristics may play a critical role in toxicity risk assessment in modern oncology settings [43,57].

## 5. Conclusions

CARG, CRASH, CRASH hematological toxicity, CARG-BC, and G8 have distinct intended purposes and evidence bases. CARG and G8 may support initial identification of patients requiring closer assessment, but low specificity limits stand-alone treatment decisions. CRASH may provide a more balanced diagnostic profile but remains supported by limited evidence, CRASH hematological toxicity has unstable and heterogeneous performance, and CARG-BC is promising for older adults with breast cancer but requires further validation. These findings apply only to the restricted set of instruments with comparable data and do not establish comparative superiority. All tools should complement, rather than replace, comprehensive geriatric assessment and individualized clinical judgment.

## Figures and Tables

**Table 1:** Characteristics of studies included.

Author/Year	Location	Study Design	Sample	Gender (M/F)	Age	Cancer Types (%)	Stage of Cancer n (%)	Scale Evaluated C
1. Zhang et al. 2019 [30]	China	Prospective Diagnostic	106	51.9/48.1	73	L: 57 (53.8) GI: 30 (28.3) B: 9 (8.5) GU: 6 (5.7) Other: 4 (3.8)	I: 5 (4.7) II: 16 (15.1) III: 26 (24.5) IV: 59 (55.7)	CARG
Zhang et al. 2019 [30]	China	Prospective Diagnostic	106	51.9/48.1	73	L: 57 (53.8) GI: 30 (28.3) B: 9 (8.5) GU: 6 (5.7) Other: 4 (3.8)	I: 5 (4.7) II: 16 (15.1) III: 26 (24.5) IV: 59 (55.7)	CRASH
Zhang et al. 2019 [30]	China	Prospective Diagnostic	106	51.9/48.1	73	L: 57 (53.8) GI: 30 (28.3) B: 9 (8.5) GU: 6 (5.7) Other: 4 (3.8)	I: 5 (4.7) II: 16 (15.1) III: 26 (24.5) IV: 59 (55.7)	CRASH hem
2. Chan et al. 2020 [31]	China	Prospective Diagnostic	259	59.3/40.7	73.4	GI: 140 (54.1) L/T: 72 (27.8) B: 20 (7.7) P: 10 (3.9) Others: 17 (6.5)	I: 3 (1.2) II: 12 (4.6) III: 75 (29) IV: 169 (65.2)	G8
Chan et al. 2020 [31]	China	Prospective Diagnostic	259	59.3/40.7	73.4	GI: 140 (54.1) L/T: 72 (27.8) B: 20 (7.7) P: 10 (3.9) Others: 17 (6.5)	I: 3 (1.2) II: 12 (4.6) III: 75 (29) IV: 169 (65.2)	CARG
3. Yoshida et al. 2020 [27]	Japan	Prospective Diagnostic	34	0/100	65	O: 20 (59) E: 14 (41)	I: 12 (35) II: 3 (9) III: 12 (35) IV: 7 (21)	CARG
4. Alibhai et al. 2021 [25]	Canada	Prospective Diagnostic	175	100/0	Chemotherapy: 73.6 A/E: 75.9	P: 175 (100%)	Metastasis: all	CARG
5. Ostwal et al. 2021 [34]	India	Prospective Diagnostic	270	149/121	69	GI: 212 (79)	I–III: 270 (100)	CARG
6. Cavdar et al. 2022 [13]	Turkey	Prospective Diagnostic	221	132/76	70.4	L: 83 (39.9) GI: 60 (28.8) B: 22 (10.6) GU: 21 (10.1) GYN: 14 (6.7)	I: 2 (1.0) II: 8 (3.8) III: 42 (20.2) IV: 156 (75)	CARG G8
7. Dhir et al. 2022 [23]	Canada	Prospective Diagnostic	30	13/17	78.1	DLB: 17 (57) CLL/ALL: 6 (20) F: 2 (7) LGB: 2 (7) HL: 2 (7) T-cell: 1 (3)	I/II: 8 (27) III/IV: 19 (63)	CARG CRASH
8. Suto et al. 2022 [29]	Japan	Retrospective Diagnostic	76	44/32	71	B: 7 (9) L: 6 (8) GI:41 (54) GYN: 3 (4) GU: 1 (1)	I: 2 (3) II: 2 (3) III: 8 (10) IV: 63 (83)	CARG
9. Ameri et al. 2023 [32]	Iran	Prospective Diagnostic	76	45/31	68.5	H and N: 24 (31.6) GI: 17 (22.3) B:10 (13.1) GYN: 7 (9.2) L: 6 (7.9) Other: 12 (15.8)	I: 3 (3.9) II:12 (15.8) III: 15 (19.7) IV: 46 (60.5)	CARG
10. Frelaut et al. 2023 [39]	USA	Prospective Diagnostic	248	138/110	80.5	CR: 53 (21) GI: 69 (28) B: 37 (15) GU: 68 (27) Other: 21 (9)	-	CARG CRASH hem
11. Lewis et al. 2023 [24]	Canada	Prospective Diagnostic	117	60/57	72.5	GI: 55 (47) B: 18 (15.4) L: 22 (18.8) GU/GYN: 12 (10.3) Other: 10 (8.5)	I: 11 (9.4) II: 25 (21.4) III: 30 (25.6) IV: 43 (36.8) Localized NOS: 8 (6.8)	CARG
12. Uchiyama et al. 2024 [28]	Japan	Prospective Diagnostic	165	130/35	77.5	L: 92 (56) GI: 62 (38) Other: 11 (6)	I: 10 (6) II: 16 (10) III: 38 (23) IV: 101 (61)	CARG
13. Hurria et al. 2011 [36]	USA	Prospective Diagnostic	500	219/281	73	L: 143 (29) GI: 135 (27) GYN: 85 (17) B: 57 (11) GU: 50 (10) Other: 30 (6)	I: 23 (5) II: 59 (12) III: 111 (22) IV: 307 (61)	CARG
14. Lagarto et al. 2026 [40]	Portugal	Retrospective Not Diagnostic	283	124/159	73	GI: 161 (56.9) GU: 49 (17.3) B: 34 (12) GYN: 24 (8.5) Other: 15(5.3)	Metastasis: 167 (59)	CARG
15. Chang et al. 2025 [42]	Taiwan	Retrospective Diagnostic	258	131/127	70	B: 56 (17) GI: 154 (46) L: 19 (6) GYN: 2 (1) Other: 17 (6.6)	I: 17 (7) II: 70 (27) III: 98 (38) IV: 73 (28)	CARG
16. Rosko et al. 2025 [35]	USA	Prospective Diagnostic	97	56/21	70	Leukemia:34 (35) Lymphoma: 29 (30) DLB: 28 (29) CLL: 6 (6)		CARG
17. Extermann et al. 2012 [38]	USA	Diagnostic	331	165/166	76	L: 71 (21.5) B: 71 (21.5) LYM: 47 (14.2) GI: 41 (12.4) Other: 101 (30.5)	I: 3 (6.3) II: 11 (14.5) III: 17 (23.6) IV: 69 (55.6)	CRASH
18. Mittal et al. 2021 [33]	India	Prospective Diagnostic	100	56/44	68.5	GI: 27 (27) GYN: 21 (21) B: 14 (14) L: 13 (13) Other: 25 (25)	I: 3 (3) II: 11 (11) III: 17 (17) IV: 69 (69)	CRASH
19. Oiwa et al. 2020 [26]	Japan	Retrospective Not Diagnostic	398	194	77	LYM: 398	III/IV: 272 (68.3)	G8
20. Magnuson et al. 2020 [37]	USA	Prospective Diagnostic	283	3/280	70.3	B: 283	I: 110 (38.9) II: 115 (40.6) III: 58 (20.5)	CARG-BC
21. Pathak et al. 2026. [22]	Canada	Retrospective Diagnostic	243	1/242	70	B: 243	I: 45 (18.5) II: 120 (49.4) III: 17 (6.9) IV: 78 (32.1)	CARG-BC

B: breast; CLL: Chronic lymphocytic; DLB: Diffuse-large B cell; E: Endometrial; F: Follicular; GI: upper gastrointestinal; GU: genitourinary; GYN: gynecologic; H: head; HL: Hodgkin Lymphoma; LGB: Low-grade B-cell; L: lung; N: neck; O: Ovarian; P: Prostate; T: thorax; ALL: Acute lymphocytic leukemia, LYM: Lymphoma.

**Table 2:** Summary of findings (SoF) to evaluate the certainty of the evidence using the GRADE methodology.

Scale	Number of Studies (Participants)	Summary of Sensitivity (95% CI)	Summary of Specificity (95% CI)	AUC	Certainly of the Evidence ^a^	Consequences in a Fictious Population of 1000 Patients ^b^				
						Assumed prevalence	True positives (95% CI)	False negatives (95% CI)	True negatives (95% CI)	False positive (95% CI)
CARG	16 (2915)	0.797 (0.716–0.859)	0.383 (0.291–0.484)	0.632	Low	52.6%	419	107	182 (138–229)	292 (245–336)
CRASH	4 (567)	0.869 (0.582–0.969)	0.682 (0.264–0.927)	0.866	Very low	52.6%	457 (306–510)	69 (16–220)	323 (125–439)	151 (35–349)
CRASH HEM	3 (685)	0.58 (0.46–0.70)	0.69 (0.24–0.94)	0.694	Very low	33.9%	257 (112–323)	82 (16–227)	351	310
G8	3 (878)	0.695 (0.611–0.768)	0.415 (0.146–0.746)	0.666	Very low	52.6%	3666 (321–404)	160 (12–205)	197 (69–354)	277 (120–405)
CARG–BC	2 (526)	0.833 (0.752–0.892)	0.544 (0.434–0.65)	0.763	Low	52.6%	438 (396–469)	88 (57–130)	258 (206–308)	216 (166–268)

^a^ Explanation of the certainty of the evidence: 1. Very high risk of bias, 2. very high inconsistency, 3. high imprecision, 4. high inconsistency. ^b^ A fictitious population is assumed for each scale. Abbreviations: AUC, area under the curve; CARG, Cancer and Aging Research Group; CARG-BC, Cancer and Aging Research Group–Breast Cancer; CI, confidence interval; CRASH, Chemotherapy Risk Assessment Scale for High-Age Patients; G8, Geriatric 8 screening tool; GRADE, Grading of Recommendations Assessment, Development and Evaluation.

## Data Availability

Additional data related to this paper may be requested from the authors.

## Supplementary Materials

The following supporting information can be downloaded at: https://www.mdpi.com/article/10.3390/cancers18152478/s1, Table S1. Preferred Reporting Items for a Systematic Review and Meta-analysis of Diagnostic Test Accuracy Studies. Table S2. The adjusted search terms as per searched electronic databases. Table S3. Reason for study exclusion. Table S4. Characteristics Toxicity of scales assessing chemotherapy toxicity. Table S5. Analysis of QUADAS-2. Figure S1. Sensitivity meta-analysis of the G8 scale. Figure S2. Specificity meta-analysis of the G8 scale. Figure S3. sROC curve of the G8 scale. Figure S4. Sensitivity meta-analysis of the CARG-BC scale. Figure S5. Specificity meta-analysis of the CARG-BC scale. Figure S6. sROC curve of the CARG-BC scale. Figure S7. Heterogeneity and Meta-Regression Analyses. Figure S8. Relationship between diagnostic study and sensitivity. Figure S9. Relationship between stage and sensitivity. Figure S10. Relationship between region and sensitivity. Figure S11. Relationship between bias risk and sensitivity. Figure S12. Funnel plot diagnostics: CARG.

## Abbreviations

The following abbreviations are used in this manuscript:

AUC	Area Under the Curve
CARG	Cancer and Aging Research Group
CI	Confidence Interval
CRASH	Chemotherapy Risk Assessment Scale for High-Age Patients
CTCAE	Common Terminology Criteria for Adverse Events
GRADE	Grading of Recommendations Assessment, Development and Evaluation
PRO-CTCAE	Patient-Reported Outcomes version of the CTCAE
ROC	Receiver Operating Characteristic
SROC	Summary Receiver Operating Characteristic
SoF	Summary of Findings
DTA	Diagnostic Test Accuracy
REML	Restricted Maximum Likelihood
CARG-BC	Cancer and Aging Research Group–Breast Cancer
