# Supplementary material for: Predictive Accuracy of Chemotherapy Toxicity Tools in Older Adults with Cancer: A Systematic Review and Diagnostic Test Accuracy Meta-Analysis
Source: Cancers (Basel). 2026 Aug 2;18(15):2478. doi: 10.3390/cancers18152478 (PMC13463990; doi:10.3390/cancers18152478)

Table S1. Preferred Reporting Items for a Systematic Review and Meta-analysis of Diagnostic Test Accuracy Studies.

Section/topic	#	PRISMA-DTA Checklist Item	Reported on page #
**TITLE / ABSTRACT**			
Title	1	Identify the report as a systematic review (+/- meta-analysis) of diagnostic test accuracy (DTA) studies.	x
Abstract	2	Abstract: See PRISMA-DTA for abstracts.	x
**INTRODUCTION**			
Rationale	3	Describe the rationale for the review in the context of what is already known.	x
Clinical role of index test	D1	State the scientific and clinical background, including the intended use and clinical role of the index test, and if applicable, the rationale for minimally acceptable test accuracy (or minimum difference in accuracy for comparative design).	x
Objectives	4	Provide an explicit statement of question(s) being addressed in terms of participants, index test(s), and target condition(s).	x
**METHODS**			
Protocol and registration	5	Indicate if a review protocol exists, if and where it can be accessed (e.g., Web address), and, if available, provide registration information including registration number.	x
Eligibility criteria	6	Specify study characteristics (participants, setting, index test(s), reference standard(s), target condition(s), and study design) and report characteristics (e.g., years considered, language, publication status) used as criteria for eligibility, giving rationale.	x
Information sources	7	Describe all information sources (e.g., databases with dates of coverage, contact with study authors to identify additional studies) in the search and date last searched.	x
Search	8	Present full search strategies for all electronic databases and other sources searched, including any limits used, such that they could be repeated.	x
Study selection	9	State the process for selecting studies (i.e., screening, eligibility, included in systematic review, and, if applicable, included in the meta-analysis).	x
Data collection process	10	Describe method of data extraction from reports (e.g., piloted forms, independently, in duplicate) and any processes for obtaining and confirming data from investigators.	x
Definitions for data extraction	11	Provide definitions used in data extraction and classifications of target condition(s), index test(s), reference standard(s) and other characteristics (e.g. study design, clinical setting).	x
Risk of bias and applicability	12	Describe methods used for assessing risk of bias in individual studies and concerns regarding the applicability to the review question.	x
Diagnostic accuracy measures	13	State the principal diagnostic accuracy measure(s) reported (e.g. sensitivity, specificity) and state the unit of assessment (e.g. per-patient, per-lesion).	x
Synthesis of results	14	Describe methods of handling data, combining results of studies and describing variability between studies. This could include, but is not limited to: a) handling of multiple definitions of target condition. b) handling of multiple thresholds of test positivity, c) handling multiple index test readers, d) handling of indeterminate test results, e) grouping and comparing tests, f) handling of different reference standards	x

Section/topic	#	PRISMA-DTA Checklist Item	Reported on page #
Meta-analysis	D2	Report the statistical methods used for meta-analyses, if performed.	x

Additional analyses	16	Describe methods of additional analyses (e.g., sensitivity or subgroup analyses, meta-regression), if done, indicating which were pre-specified.	x
**RESULTS**			
Study selection	17	Provide numbers of studies screened, assessed for eligibility, included in the review (and included in meta-analysis, if applicable) with reasons for exclusions at each stage, ideally with a flow diagram.	x
Study characteristics	18	For each included study provide citations and present key characteristics including: a) participant characteristics (presentation, prior testing), b) clinical setting, c) study design, d) target condition definition, e) index test, f) reference standard, g) sample size, h) funding sources	x
Risk of bias and applicability	19	Present evaluation of risk of bias and concerns regarding applicability for each study.	x
Results of individual studies	20	For each analysis in each study (e.g. unique combination of index test, reference standard, and positivity threshold) report 2x2 data (TP, FP, FN, TN) with estimates of diagnostic accuracy and confidence intervals, ideally with a forest or receiver operator characteristic (ROC) plot.	x
Synthesis of results	21	Describe test accuracy, including variability; if meta-analysis was done, include results and confidence intervals.	x
Additional analysis	23	Give results of additional analyses, if done (e.g., sensitivity or subgroup analyses, meta-regression; analysis of index test: failure rates, proportion of inconclusive results, adverse events).	x
**DISCUSSION**			
Summary of evidence	24	Summarize the main findings including the strength of evidence.	x
Limitations	25	Discuss limitations from included studies (e.g. risk of bias and concerns regarding applicability) and from the review process (e.g. incomplete retrieval of identified research).	x
Conclusions	26	Provide a general interpretation of the results in the context of other evidence. Discuss implications for future research and clinical practice (e.g. the intended use and clinical role of the index test).	x
**FUNDING**			
Funding	27	For the systematic review, describe the sources of funding and other support and the role of the funders.	x

Adapted From: McInnes MDF, Moher D, Thombs BD, McGrath TA, Bossuyt PM, The PRISMA-DTA Group (2018). Preferred Reporting Items for a Systematic Review and Meta-analysis of Diagnostic Test Accuracy Studies: The PRISMA-DTA Statement. JAMA. 2018 Jan 23;319(4):388-396. doi: 10.1001/jama.2017.19163.

Table S2. The adjusted search terms as per searched electronic databases.

An update of the search was conducted until May 2026.

PubMed			
	#1	"Drug-Related Side Effects and Adverse Reactions" [TIAB] OR ("chemotherapy toxicity"[TIAB])	2,037
	#2	(Aged[TIAB] OR "Elder*"[TIAB]) OR "Frail elderly"[TIAB] OR ("Aged, 80 and over"[TIAB] OR "Oldest old"[TIAB] OR "older"[TIAB])	1,468,623
	#3	#1 AND #2	333
Scopus			
	#1	TITLE ("Drug-Related Side Effects and Adverse Reactions" OR "chemotherapy toxicity")	179
	#2	TITLE (aged OR elder* OR "Frail elderly" OR "Aged, 80 and over" OR "Oldest old" OR older)	475,933
	#3	#1 AND #2	39
Embase			
	#1	'Drug-Related Side Effects and Adverse Reactions':ti,ab OR 'chemotherapy toxicity':ti,ab	1391
	#2	Aged:ti,ab OR Elder*:ti,ab OR 'Frail elderly':ti,ab OR 'Aged, 80 and over':ti,ab OR 'Oldest old':ti,ab OR older:ti,ab	2,026,234
	#3	#1 AND #2	493
Web of Science			
	#1	TI=("Drug-Related Side Effects and Adverse Reactions" OR "chemotherapy toxicity")	227
	#2	TI=(Aged OR Elder* OR "Frail elderly" OR "Aged, 80 and over" OR "Oldest old" OR older)	1,064,043
	#3	#1 AND #2	82
ScienceDirect			
	#1	Title, abstract, keywords: ("Drug-Related Side Effects and Adverse Reactions" OR "chemotherapy toxicity")	683
	#2	Title, abstract, keywords: ("Aged" OR "Elder" OR "Frail elderly" OR "Aged, 80 and over" OR "Oldest old" OR "older")	242,813
	#3	#1 AND #2	288
Google Scholar			
	#1	allintitle: "Drug-Related Side Effects and Adverse Reactions" OR "chemotherapy toxicity"	744
	#2	allintitle: "Aged" OR "Elder*" OR "Frail elderly" OR "Aged, 80 and over" OR "Oldest old" OR "older"	386,000
	#3	#1 AND #2	254
Virtual Health Library (VHL)			
	#1	ti: ("Drug-Related Side Effects" OR "Adverse Reactions" OR "chemotherapy toxicity" OR "pharmacotherapy side effects")	5131
	#2	ti: ("Aged" OR "Elderly" OR "Elder*" OR "Frail elderly" OR "Aged, 80 and over" OR "Oldest old" OR "older")	390,559
	#3	#1 AND #2	104
TOTAL		PubMed (n =333); Scopus (n=39); Web of Sciences(n=82); Embase (n=493); ScienceDirect (288); Google Scholar (254) and VHL (104)	1,593
Duplicates			666
			927

The search was expanded with a second strategy until July 2026.

PubMed	
#1 ((("Antineoplastic Combined Chemotherapy Protocols"[Mesh] OR "Antineoplastic Agents"[Mesh] OR chemotherap*[tiab] OR systemic anticancer therap*[tiab] OR systemic anti-cancer therap*[tiab] OR antineoplastic treatment*[tiab] OR anticancer treatment*[tiab]))	1,071,070
#2 (("Drug-Related Side Effects and Adverse Reactions"[Mesh] OR toxicit*[tiab] OR adverse event*[tiab] OR adverse effect*[tiab] OR treatment-related toxicit*[tiab] OR chemotherapy-related toxicit*[tiab] OR severe toxicit*[tiab] OR grade 3 toxicit*[tiab] OR grade 4 toxicit*[tiab] OR grade 5 toxicit*[tiab])))	1,213,160
#3 (("CARG"[tiab] OR "Cancer and Aging Research Group"[tiab] OR "Cancer and Ageing Research Group"[tiab] OR "CRASH"[tiab] OR "Chemotherapy Risk Assessment Scale for High-Age Patients"[tiab] OR "CARG-BC"[tiab] OR "Geriatric 8"[tiab] OR "G8"[tiab] OR "ACTTOP"[tiab] OR "Anti-Cancer Treatment Toxicity in Older Patients"[tiab] OR "MOST"[tiab] OR "Kanazu"[tiab] OR "CARG-8"[tiab]))	17,663
#1 AND #2 AND #3	162

Table S3. Reason for study exclusion.

Author/Year of publication	Study name	Exclusion reason
Ilie et al. 2012	The Usefulness of G8 Evaluation in Therapeutic Decision and Prediction of Tolerance in Locally Advanced or Metastatic Squamous Cell Head and Neck Cancer (SCCHN) Patients Older Than 65 Years	Conference abstract or scientific poster
Broughman et al. 2015	Prevalence of sarcopenia in older patients with colorectal cancer	Does not evaluate criteria of included tools
Chamarthy et al. 2020	Use of CARG toxicity tool to predict chemotherapy toxicity in older veterans to facilitate better treatment outcomes	Conference abstract or scientific poster
Frelaut et al. 2020	External validation of two predictive scores of chemotherapy toxicities among older patients with solid cancer, from ELCAPA prospective cohort	Conference abstract or scientific poster
Rosko et al. 2021	Evaluating the Carg Chemotherapy Toxicity Calculator Among Older Adults Newly Diagnosed with Hematologic Malignancy	Conference abstract or scientific poster
AlSendi et al. 2024	Pilot study of the implementation of G8 screening tool, Cognitive screening assessment and Chemotherapy Toxicity assessment in older adults with cancer in a Tertiary University Hospital in Ireland	It is a prevalence study, but does not evaluate the characteristics of the tools included
Chang et al. 2024	Validation of a chemotherapy toxicity prediction model in older adults with cancer in Taiwan	Conference abstract or scientific poster

Moth et al. 2019	Predicting chemotherapy toxicity in older adults: Comparing the predictive value of the CARG Toxicity Score with oncologists' estimates of toxicity based on clinical judgement	Not data to assess diagnostic performance.
Jayani et al. 2020	Association between a cognitive screening test and severe chemotherapy toxicity in older adults with cancer	Not diagnostic assessment design
Lok Chan et al. 2021	The predictive value of G8 and the Cancer and aging research group chemotherapy toxicity tool in treatment-related toxicity in older Chinese patients with cancer	Not data to assess diagnostic performance.
Baitar et al. 2014	Geriatric screening results and the association with severe treatment toxicity after the first cycle of (radio)chemotherapy	Not diagnostic assessment design
Bergerot et al. 2019	Association between chemotherapy toxicity risk scores and physical symptoms among older Brazilian adults with cancer	Not diagnostic assessment design
Dotan et al. 2020	Associations Between Nutritional Factors and Chemotherapy Toxicity in Older Adults With Solid Tumors	Not diagnostic assessment design
Nishijima et al. 2022	Impact of the Cancer and Aging Research Group score and treatment intensity on survival and toxicity outcomes in older adults with advanced noncolorectal gastrointestinal cancers	Not diagnostic assessment design
Bergerot et al. 2022	Implementation and evaluation of a remote geriatric assessment and intervention program in Brazil	Not diagnostic assessment design

Mariano et al. 2019	Utility of a chemotherapy toxicity prediction<br>tool for older patients in a community setting	Not data to assess diagnostic performance.
Suto et al. 2022	Predicting Chemotherapy-Related Adverse Events in Elderly Cancer Patients with Prior Anticancer Therapy	Secondary data from another study that has been selected.
Hamid et al. 2022	Chemotherapy Toxicity in Older Adults Optimized by Geriatric Assessment and Intervention: A Non-Comparative Analysis	Not diagnostic assessment design
Ji et al. 2023	Toxicity risk score and clinical decline after adjuvant chemotherapy in older breast cancer survivors	Not diagnostic assessment design

Table S4. Characteristics Toxicity of scales assessing chemotherapy toxicity.

Author/Year	TP	FN	FP	TN	Toxicity N (%)	Sensitivity	Specificity
1. Zhang et al. 2019 **CARG**	53	1	37	15	54 (50.9)	98.1%	28.8%
Zhang et al. 2019 **CRASH**	13	13	9	71	26 (24.5)	50%	88.8%
Zhang et al. 2019 **CRASH hem**	5	3	14	84	8 (7.6)	63.6%	80%
2. Chan et al. 2020 **G8**	102	44	87	26	146 (56.3)	69.9%	21.6%
Chan et al. 2020 **CARG**	98	48	74	39	146 (56.3)	52.6%	42.6%
3. Yoshida et al. 2020 **CARG**	13	11	2	8	24 (58)	54.2%	80%
4. Alibhai et al. 2021 **CARG**	28	10	12	21	38 (53.5)	70%	67.7%
5. Ostwal et al. 2021 **CARG**	110	30	88	42	140 (51.9)	78.6%	32.3%
6. Cavdar et al. 2022 **CARG**	95	8	49	56	103 (49.5)	92.2 %	53.3 %
Cavdar et al. 2022 **G8**	88	25	68	27	113 (54.3)	77.9 %	28.4 %
7. Dhir et al. 2022 **CARG**	12	1	13	4	13 (43.3)	92.3 %	23.5 %
Dhir et al. 2022 **CRASH**	12	1	5	12	13 (43.3)	92.3 %	70.6 %
8. Suto et al. 2022 **CARG**	32	10	18	16	42 (55.3)	76.2 %	47.1 %
9. Ameri et al. 2023 **CARG**	14	4	36	22	18 (23.7)	77.8%	37.9%
10. Frelaut et al. 2023 **CARG**	129	9	91	2	138 (59.7)	93.5%	2.2%
Frelaut et al. 2023 **CRASH hem**	49	41	41	34	90 (54.5)	54.4%	45.3%
11. Lewis et al. 2023 **CARG**	58	9	36	14	67 (57.3)	86.6%	28%
12. Uchiyama et al. 2024 **CARG**	95	57	8	5	152 (92.1)	62.5%	38.5%
13. Hurria et al. 2011 **CARG**	199	44	121	100	243 (52.4)	81.9%	45.2%

14. Lagarto et al. 2026 **CARG**	111	14	110	158	125 (31.8)	88.8%	59%
15. Chang et al. 2025 **CARG**	90	33	83	52	123 (47.7)	73.2%	38.5%
16. Rosko et al. 2025 **CARG**	40	42	26	9	82 (70.1)	48.8%	25.7%
17. Extermann et al. 2012 **CRASH**	195	17	102	17	212 (64)	92%	14.3%
Extermann et al. 2012 **CRASH hem**	101	5	173	52	106 (32)	95.3%	23.1%
18. Mittal et al. 2021 **CRASH**	44	0	6	90	44 (31.4)	100%	93.8%
19. Oiwa et al. 2020 **G8**	150	92	53	157	242 (53.5)	62%	74.8%
20. Magnuson et al. 2020 **CARG-BC**	113	26	45	70	139 (54.7)	81.3%	60.9%
21. Pathak et al. 2026 **CARG-BC**	105	26	45	67	131 (53.9)	80.2%	59.8%

TP: true positive; FN: false negative; FP: false positive; TN: true negative; NR: not reported.

Table S5. Analysis of QUADAS-2

Study	Risk of bias				Applicability concerns		
	Patient selection	Index test	Reference standard	Flow and timing	Patient selection	Index test	Reference standard
Zhang et al. 2019	Unclear	Low	Low	Low	Low	Low	Low
Chan et al. 2020	Low	Low	Low	Low	Low	Low	Low
Yoshida et al. 2020	Low	Low	Low	Low	Low	Low	Low
Alibhai et al. 2021	Low	Low	Low	High	Low	Low	Low
Ostwal et al. 2021	Low	Low	Low	Low	Low	Low	Low
Cavdar et al. 2022	Low	Low	Low	Low	Low	Low	Low
Dhir et al. 2022	Low	Low	Low	High	Low	Low	Low
Suto et al. 2022	Low	Unclear	Low	Low	Low	Low	Low
Ameri et al. 2023	Low	Low	Low	High	Low	Low	Low
Frelaut et al. 2023	Low	High	Low	High	Low	High	Low
Lewis et al. 2023	Low	Unclear	Low	High	Low	Low	Low
Uchiyama et al. 2024	Unclear	Low	Low	Low	Low	Low	Low
Hurria et al. 2011	Low	Unclear	Low	Low	Low	Unclear	Low
Lagarto et al. 2026	Low	High	Low	High	Low	High	Low
Chang et al. 2025	Low	Low	Low	High	Low	Low	Low

Rosko et al. 2025	Low	Low	Low	Low	Low	Low	Low
Extermann et al. 2012	Low	Low	Unclear	Unclear	Low	Low	Unclear
Mittal et al. 2021	Low	Low	Low	Low	Unclear	Low	Low
Oiwa et al. 2020	Low	Low	High	High	Low	Low	High
Magnuson et al. 2020	Low	Low	Low	Unclear	Low	Low	Low
Pathak et al. 2026	Low	Low	Low	High	Low	Low	High

Figure S1. Sensitivity meta-analysis of the G8 scale.

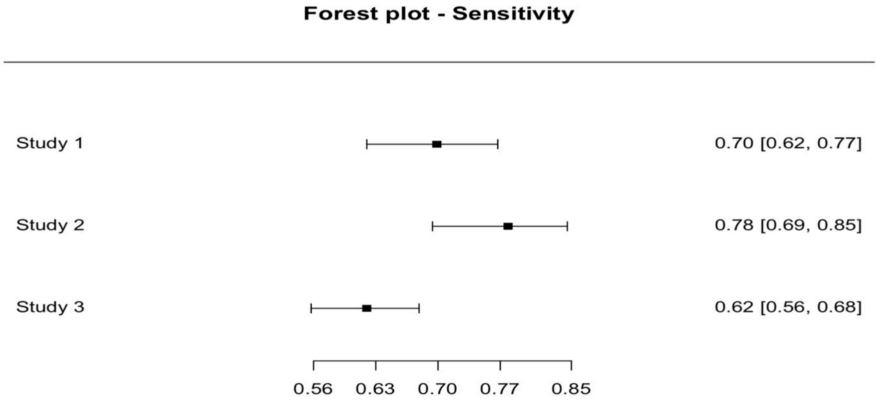

Figure S2. Specificity meta-analysis of the G8 scale

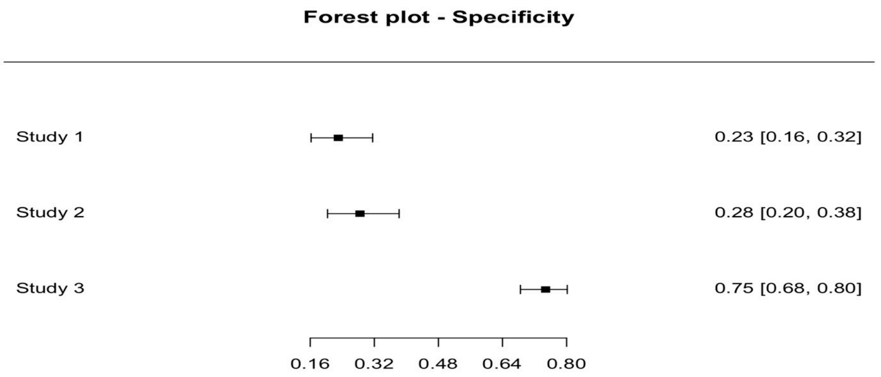

Figure S3. sROC curve of the G8 scale

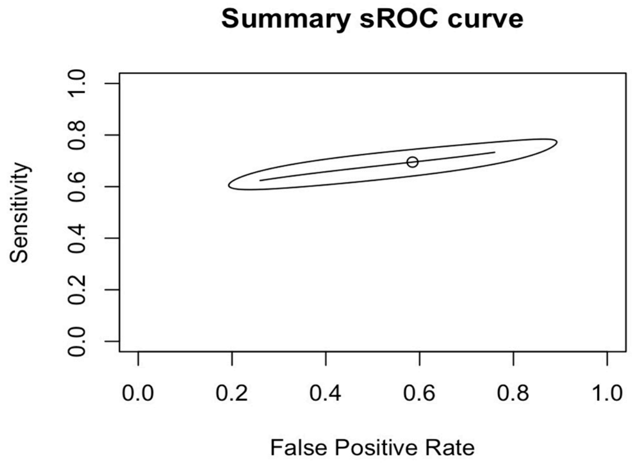

Figure S4. Sensitivity meta-analysis of the CARG-BC scale

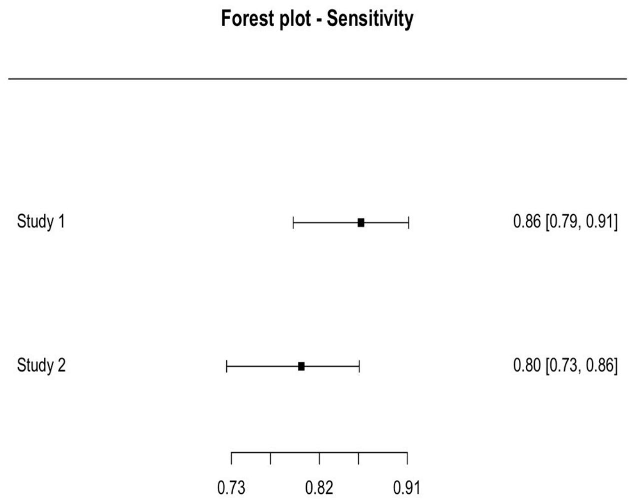

Figure S5. Specificity meta-analysis of the CARG-BC scale

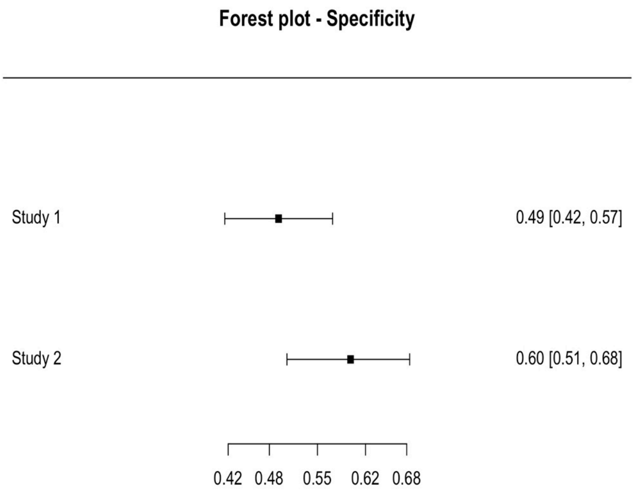

Figure S6. sROC curve of the CARG-BC scale

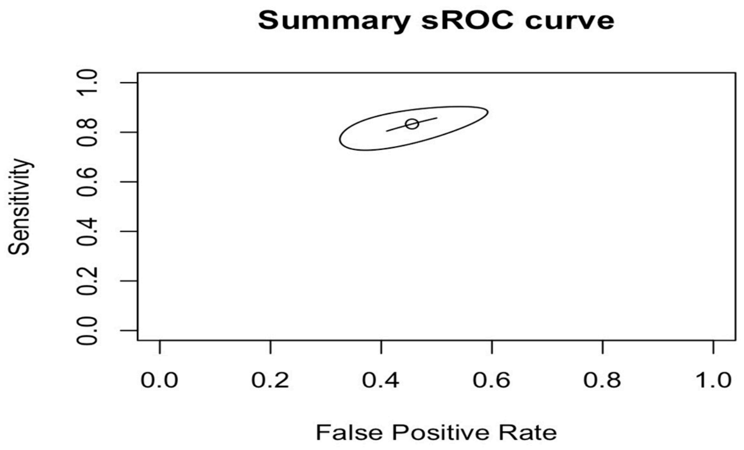

Figure S7. Heterogeneity and Meta-Regression Analyses

```
Call:  reitsma.default(data = df, formula = cbind(tsens, tfpr) ~ age)

Bivariate diagnostic random-effects meta-analysis
Estimation method: REML

Fixed-effects coefficients
                    Estimate Std. Error      z Pr(>|z|) 95%ci.lb 95%ci.ub
tsens.(Intercept)     -5.653      4.205 -1.344    0.179  -13.896    2.589
tsens.age              0.097      0.058  1.669    0.095   -0.017    0.211 .
tfpr.(Intercept)     -10.251      4.231 -2.423    0.015  -18.543   -1.959 *
tfpr.age               0.149      0.059  2.538    0.011    0.034    0.263 *
---
Signif. codes:  0 '***' 0.001 '**' 0.01 '*' 0.05 '.' 0.1 ' ' 1

Variance components: between-studies Std. Dev and correlation matrix
      Std. Dev tsens  tfpr
tsens    0.762 1.000     .
tfpr     0.660 0.094 1.000

 logLik     AIC     BIC
 16.139 -18.278  -8.017


I2 estimates
Zhou and Dendukuri approach:  0.3 %
Holling sample size unadjusted approaches:  64.6 - 83.5 %
Holling sample size adjusted approaches:  3.5 - 6.4 %
```

```
Call:  reitsma.default(data = df, formula = cbind(tsens, tfpr) ~ region)

Bivariate diagnostic random-effects meta-analysis
Estimation method: REML

Fixed-effects coefficients
                  Estimate Std. Error      z Pr(>|z|) 95%ci.lb 95%ci.ub
tsens.(Intercept)    1.571      0.344  4.563    0.000    0.896    2.246 ***
tsens.region        -0.357      0.459 -0.778    0.437   -1.256    0.542
tfpr.(Intercept)     0.701      0.340  2.060    0.039    0.034    1.368   *
tfpr.region         -0.380      0.450 -0.845    0.398   -1.262    0.502
---
Signif. codes:  0 ‘***’ 0.001 ‘**’ 0.01 ‘*’ 0.05 ‘.’ 0.1 ‘ ’ 1

Variance components: between-studies Std. Dev and correlation matrix
      Std. Dev tsens  tfpr
tsens    0.823 1.000     .
tfpr     0.813 0.318 1.000

 logLik     AIC     BIC
 16.699 -19.398  -9.137


I2 estimates
Zhou and Dendukuri approach:  50.9 %
Holling sample size unadjusted approaches:  64.6 - 83.5 %
Holling sample size adjusted approaches:  3.5 - 6.4 %
```

```
Call:  reitsma.default(data = df, formula = cbind(tsens, tfpr) ~ sample)

Bivariate diagnostic random-effects meta-analysis
Estimation method: REML

Fixed-effects coefficients
                  Estimate Std. Error      z Pr(>|z|) 95%ci.lb 95%ci.ub
tsens.(Intercept)    1.037      0.439  2.364    0.018    0.177    1.896 *
tsens.sample         0.002      0.002  0.864    0.388   -0.002    0.006
tfpr.(Intercept)     0.500      0.429  1.165    0.244   -0.341    1.341
tfpr.sample         -0.000      0.002 -0.034    0.973   -0.004    0.004
---
Signif. codes:  0 ‘***’ 0.001 ‘**’ 0.01 ‘*’ 0.05 ‘.’ 0.1 ‘ ’ 1

Variance components: between-studies Std. Dev and correlation matrix
      Std. Dev tsens  tfpr
tsens    0.802 1.000     .
tfpr     0.812 0.349 1.000

logLik    AIC    BIC
 5.700  2.601 12.861


I2 estimates
Zhou and Dendukuri approach:  0.7 %
Holling sample size unadjusted approaches:  64.6 - 83.5 %
Holling sample size adjusted approaches:  3.5 - 6.4 %
```

```
Call:  reitsma.default(data = df[!is.na(df$stage_iv), ], formula = cbind(tsens,
    tfpr) ~ stage_iv)

Bivariate diagnostic random-effects meta-analysis
Estimation method: REML

Fixed-effects coefficients
                  Estimate Std. Error      z Pr(>|z|) 95%ci.lb 95%ci.ub
tsens.(Intercept)    0.980      0.676  1.450    0.147   -0.344    2.303
tsens.stage_iv       0.006      0.011  0.567    0.570   -0.015    0.027
tfpr.(Intercept)     0.769      0.461  1.667    0.096   -0.135    1.673 .
tfpr.stage_iv       -0.009      0.007 -1.247    0.212   -0.023    0.005
---
Signif. codes:  0 ‘***’ 0.001 ‘**’ 0.01 ‘*’ 0.05 ‘.’ 0.1 ‘ ’ 1

Variance components: between-studies Std. Dev and correlation matrix
      Std. Dev tsens  tfpr
tsens    0.715 1.000     .
tfpr     0.390 0.067 1.000

logLik    AIC    BIC
 9.224 -4.449  3.797


I2 estimates
Zhou and Dendukuri approach:  6.7 %
Holling sample size unadjusted approaches:  66.2 - 81.7 %
Holling sample size adjusted approaches:  3.6 - 5.7 %
```

```
Call:  reitsma.default(data = df, formula = cbind(tsens, tfpr) ~ dxstudy)

Bivariate diagnostic random-effects meta-analysis
Estimation method: REML

Fixed-effects coefficients
                  Estimate Std. Error      z Pr(>|z|) 95%ci.lb 95%ci.ub
tsens.(Intercept)    1.393      0.331  4.213    0.000    0.745    2.041 ***
tsens.dxstudy       -0.038      0.468 -0.080    0.936   -0.956    0.880
tfpr.(Intercept)     0.548      0.299  1.833    0.067   -0.038    1.134   .
tfpr.dxstudy        -0.146      0.444 -0.328    0.743   -1.016    0.725
---
Signif. codes:  0 ‘***’ 0.001 ‘**’ 0.01 ‘*’ 0.05 ‘.’ 0.1 ‘ ’ 1

Variance components: between-studies Std. Dev and correlation matrix
      Std. Dev tsens  tfpr
tsens    0.852 1.000     .
tfpr     0.806 0.345 1.000

 logLik     AIC     BIC
 16.229 -18.457  -8.197


I2 estimates
Zhou and Dendukuri approach:  52 %
Holling sample size unadjusted approaches:  64.6 - 83.5 %
Holling sample size adjusted approaches:  3.5 - 6.4 %
```

```
>  modelo <- reitsma(formula= cbind(tsens, tfpr) ~ biasrisk, data = df)
>  summary(modelo)
Call:  reitsma.default(data = df, formula = cbind(tsens, tfpr) ~ biasrisk)

Bivariate diagnostic random-effects meta-analysis
Estimation method: REML

Fixed-effects coefficients
                  Estimate Std. Error     z Pr(>|z|) 95%ci.lb 95%ci.ub
tsens.(Intercept)    1.104      0.278 3.971    0.000    0.559    1.649 ***
tsens.biasrisk       0.614      0.433 1.420    0.156   -0.234    1.462
tfpr.(Intercept)     0.339      0.295 1.152    0.249   -0.238    0.917
tfpr.biasrisk        0.332      0.448 0.742    0.458   -0.545    1.209
---
Signif. codes:  0 ‘***’ 0.001 ‘**’ 0.01 ‘*’ 0.05 ‘.’ 0.1 ‘ ’ 1

Variance components: between-studies Std. Dev and correlation matrix
      Std. Dev tsens  tfpr
tsens    0.762 1.000     .
tfpr     0.809 0.307 1.000

 logLik     AIC     BIC
 17.170 -20.340 -10.079


I2 estimates
Zhou and Dendukuri approach:  47.1 %
Holling sample size unadjusted approaches:  64.6 - 83.5 %
Holling sample size adjusted approaches:  3.5 - 6.4 %
[illegible]
```

Figure S8. Relationship between diagnostic study and sensitivity

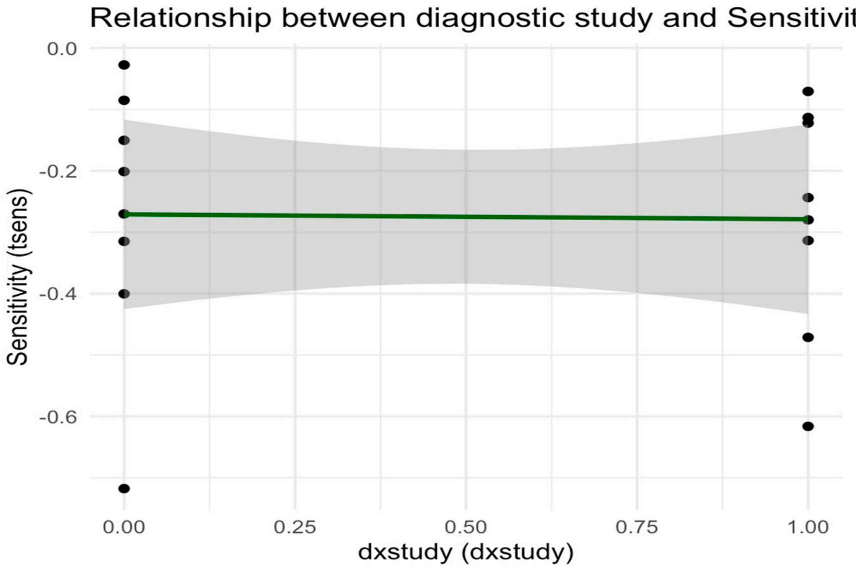

Figure S9. Relationship between stage and sensitivity

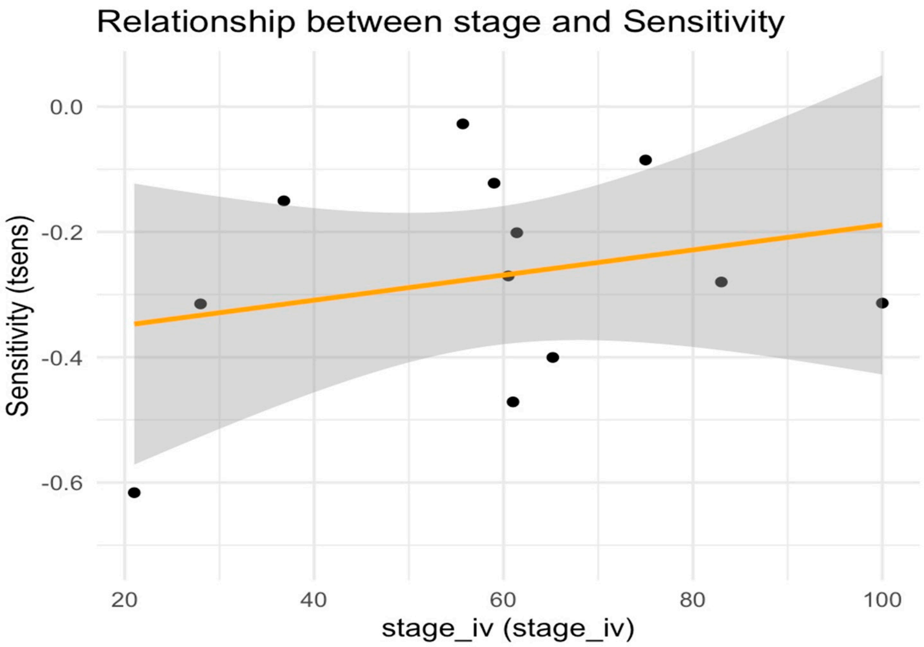

Figure S10. Relationship between region and sensitivity

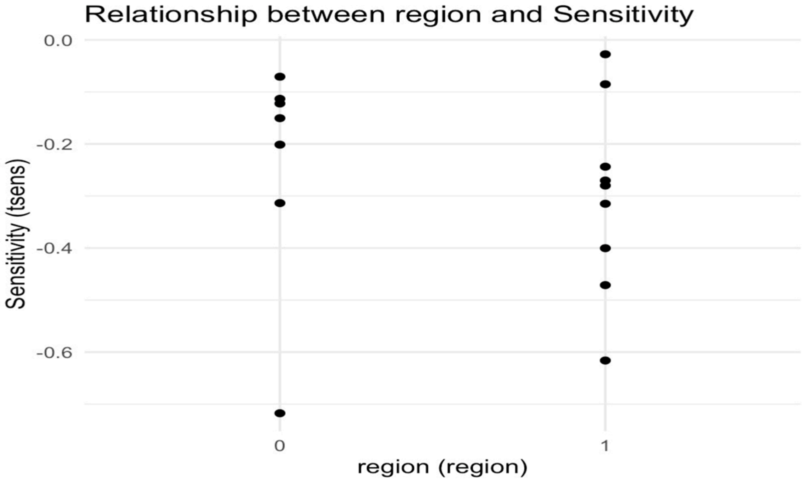

Figure S11. Relationship between bias risk and sensitivity

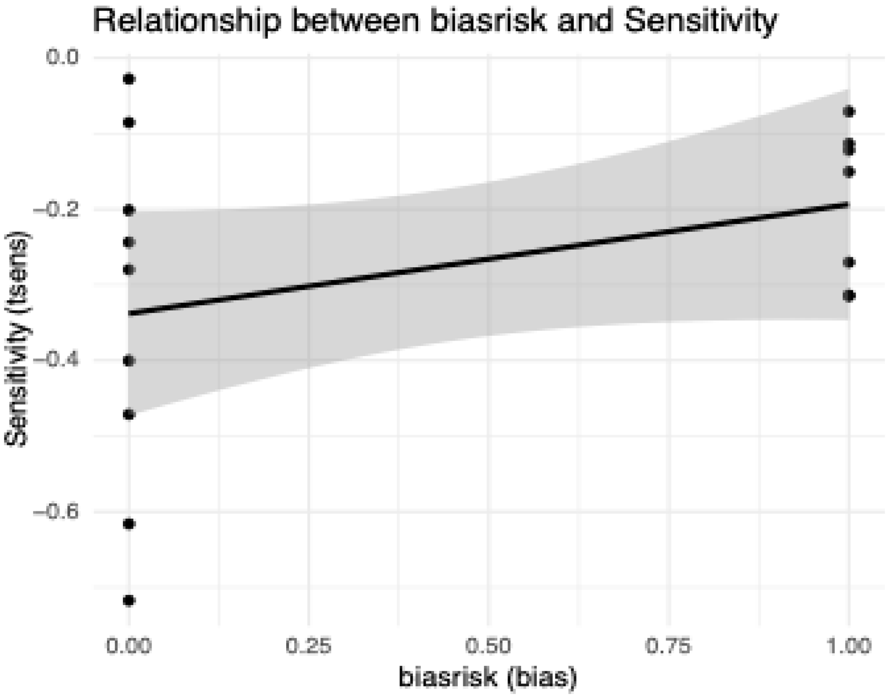

Figure S12. Funnel plot diagnostics: CARG

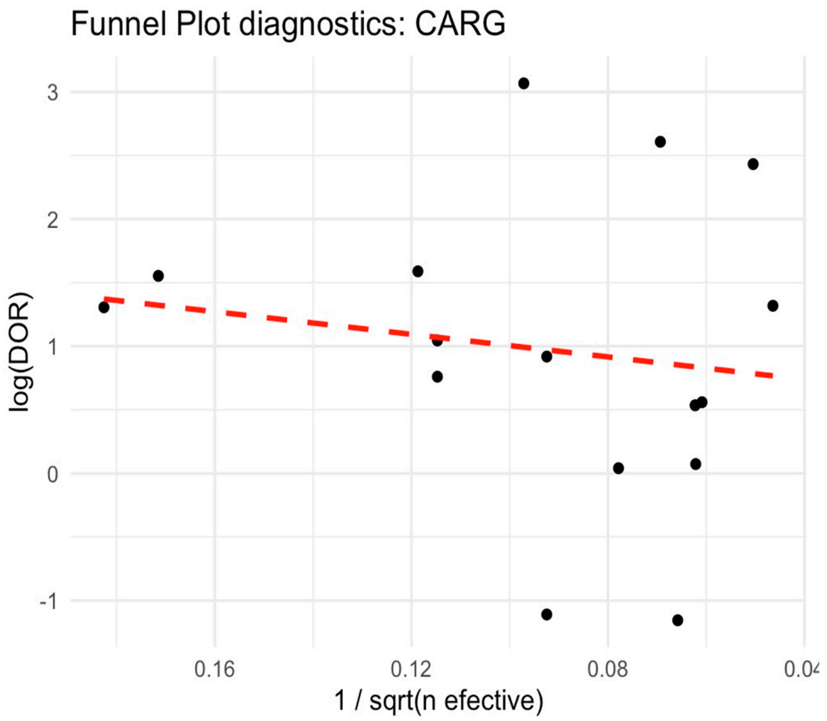

Supplement: Supplementary file 1 [file cancers-18-02478-s001.zip › cancers-4412931-supplementary.pdf]
